# Biochemical and functional characterization of glycosylation-associated mutational landscapes in colon cancer

**DOI:** 10.1038/srep23642

**Published:** 2016-03-23

**Authors:** Srividya Venkitachalam, Leslie Revoredo, Vinay Varadan, Ryan E. Fecteau, Lakshmeswari Ravi, James Lutterbaugh, Sanford D. Markowitz, Joseph E. Willis, Thomas A. Gerken, Kishore Guda

**Affiliations:** 1 Case Comprehensive Cancer Center, Case Western Reserve University, Cleveland OH 44106, U.S.A; 2 Department of Chemistry, Case Western Reserve University, Cleveland OH 44106, U.S.A; 3 Division of General-Medical Sciences-Oncology, Case Western Reserve University, Cleveland OH 44106, U.S.A; 4 Division of Hematology and Oncology, Case Western Reserve University, Cleveland OH 44106, U.S.A; 5 Department of Pathology, Case Western Reserve University, Cleveland OH 44106, U.S.A; 6 Department of Pediatrics, Case Western Reserve University, Cleveland OH 44106, U.S.A; 7 Department of Biochemistry, Case Western Reserve University, Cleveland OH 44106, U.S.A

## Abstract

The molecular basis of aberrant protein glycosylation, a pathological alteration widespread in colorectal cancers (CRC), and the mechanisms by which it contributes to tumor progression remain largely unknown. We performed targeted re-sequencing of 430 glycosylation-associated genes in a series of patient-derived CRC cell lines (N = 31) and matched primary tumor tissues, identifying 12 new significantly mutated glycosylation-associated genes in colon cancer. In particular, we observed an enrichment of mutations in genes (*B3GNT2*, *B4GALT*2, *ST6GALNAC2*) involved in the biosynthesis of *N*- and Cores 1–3 *O*-linked glycans in the colon, accounting for ~16% of the CRCs tested. Analysis of independent large-scale tumor tissue datasets confirmed recurrent mutations within these genes in colon and other gastrointestinal cancers. Systematic biochemical and phenotypic characterization of the candidate wild-type and mutant glycosyltransferases demonstrated these mutations as either markedly altering protein localization, post-translational modification, encoded enzymatic activities and/or the migratory potential of colon carcinoma cells. These findings suggest that functionally deleterious mutations in glycosyltransferase genes in part underlie aberrant glycosylation, and contribute to the pathogenesis of molecular subsets of colon and other gastrointestinal malignancies.

Protein glycosylation is a key post-translational modification that plays a fundamental role in regulating multiple cellular processes including cell adhesion, migration, cell-cell recognition and immune surveillance^1^. Glycosylation of newly synthesized peptides may be initiated in both the Endoplasmic reticulum (ER) and Golgi apparatus and is catalyzed by a series of specific glycosyltransferases that may display overlapping specificities depending on the transferase^1^,^2^,^3^,^4^. These enzymes typically transfer single sugar residues from nucleotide-sugar donors to protein and sugar acceptors, the latter resulting in glycan elongation forming a vast array of glycan structures^5^. The resulting glycans are typically characterized as *N*-linked or *O*-linked based on the amino acid residues (Asn or Ser/Thr) the glycans are attached to, which also corresponds to their origin of initiation in the ER or Golgi respectively^1^.

Aberrant protein glycosylation is a hallmark of many human cancers including colorectal cancers (CRC)^6^,^7^,^8^. However, the molecular basis of aberrant glycosylation and the mechanisms by which it contributes to tumor progression remain largely unknown. We previously reported the first finding of somatic and germline inactivating mutations in the gene encoding for GALNT12, a key enzyme involved in the initiating step of mucin type *O*-glycosylation, in a subset of colon cancer cases^9^. Our initial findings strongly suggest that mutations in *O*-glycosylation pathway genes may in part underlie aberrant protein glycosylation commonly seen in colon and other cancers, and potentially contribute to the development of a subset of these malignancies. Since both protein *N*- and *O*-glycosylation are complex processes involving a multitude of enzymes, we initiated a study to characterize the extent and significance of genetic defects in the colon cancer glycome. By employing comprehensive genomic, biochemical, and functional approaches in a series of patient-derived colon cancer cell lines and matched primary tumors, we identified significant molecular and functional defects in 3 genes that likely control the biosynthesis (termination and elongation) of *N*- and Core 1–3 *O*-linked glycans expressed in the colon, thus uncovering mechanisms potentially contributing to aberrant glycosylation and colon tumor progression.

## Results

### Catalog of somatic mutations in glycosylation pathway genes in colon cancer

We performed targeted re-sequencing of 430 glycosylation genes in a set of patient-derived microsatellite stable (MSS) CRC cell lines (N = 31) (Supplementary Tables S1–S3) to determine the type and extent of glycosylation pathway defects in colon cancer, and to assess for the prevalence of bi-allelic defects in these glycosylation pathway genes. Following sequential filtering and confirmation in antecedent primary colon tumors (see Methods), we identified a total of 41 non-silent mutations mapping to 36 unique genes, with the majority of mutations being missense alterations (Supplementary Table S4). Eighteen of the missense mutations were predicted to be deleterious in nature by SIFT and/or Polyphen algorithms^10^,^11^. Five mutations were highly likely deleterious in nature including, 3 nonsense mutations (*ALG13*, *B3GNT2* and *MAN2B2*), a splice site mutation (*ALG6*), and a frame shift deletion mutation (*ST8SIA3*) (Supplementary Table S4). Colon cancers with mutant *B4GALT2*, *MGAT2*, or *ST8SIA3* showed genomic loss of respective wild-type alleles, while colon cancers with mutant *B3GALT1*, *GAL3ST1*, *GLT25D2*, or *PIGO* showed loss of transcript expression of respective wild-type alleles, providing evidence for bi-allelic defects in these genes in colon cancer (Supplementary Table S4).

**Figure 1:**
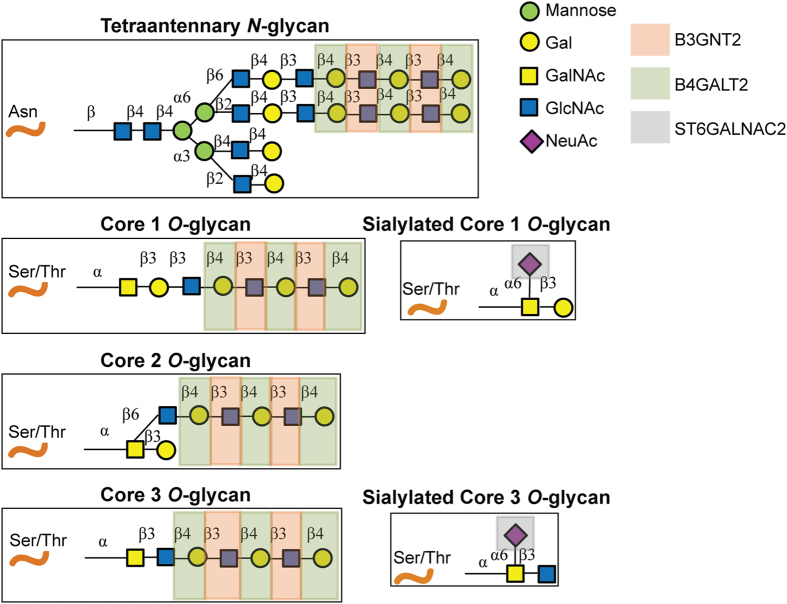
Protein glycosylation pathways involving B3GNT2, B4GALT2 and ST6GALNAC2. Synthesis of polylactosamine structures (polyLacNAc) on tetraantennary N-linked glycans and on the Core 1, 2 and 3 O-glycans, catalyzed by B3GNT2 and B4GALT2 enzymes. The addition of sialic acid (NeuNAc) to the Core 1 and 3 O-glycan chains by ST6GALNAC2 results in chain termination^15^,^26^,^33^.

We next determined which among the 36 candidate genes are mutated at a significantly higher rate than the expected background rate in the CRCs under study. Using the statistical framework as previously described by our group^12^,^13^, we identified 12 of the 36 genes to be significantly mutated in CRCs (P ≤ 0.01, FDR < 0.05) (Table 1, Supplementary Table S5). Interestingly, we observed three of these genes *B3GNT2*, *ST6GALNAC2*, and *B4GALT2* mapping to protein glycosylation pathways that are involved in the formation of polylactosamine chain extensions on *N*- and *O*-linked glycans (B3GNT2 and B4GALT2) and in the termination of *O*-glycan Core 1 and 3 structures (ST6GALNAC2) (see Fig. 1)^14^,^15^,^16^. Together, mutations in these three genes accounted for five CRC cases, with two missense and one nonsense mutation in *B3GNT2*, two missense mutations in *ST6GALNAC2*, and one missense mutation with a loss of the wild-type allele in *B4GALT2* (Table 2, Supplementary Table S5). Furthermore, *in silico* prediction by SIFT and/or PolyPhen revealed four of the five missense mutations within these genes to significantly alter protein function (Table 2). Mutual exclusivity analysis showed that mutations affecting *B3GNT2*, *ST6GALNAC2*, *B4GALT2* individually, or any of the 36 candidate glycosylation genes as a group, as not being independent of known driver oncogenic mutations in *KRAS* or *BRAF* in colon cancer (Supplementary Table 4, Supplementary Fig. 1), indicating that the glycosylation defects may play a complementary role to other mitogenic signaling pathways in the multi-step colon cancer progression model. Evaluation of independent large-scale cancer datasets^17^,^18^ revealed recurrent somatic mutations in *B3GNT2*, *B4GALT2* and *ST6GALNAC2*, accounting for ~3% of CRC cases (Supplementary Table S6). These findings suggest that genetic defects in glycosyltransferases involved in the biosynthesis of Core 1–3 *O*-glycans potentially contribute to the pathogenesis of molecular subsets of gastrointestinal cancers.

### Biochemical characterization of wild-type and mutant glycosyltransferases

As suggested by our previous findings in GALNT12^9^, mutations in glycosylation-associated genes could alter enzymatic activity of the encoded glycosyltransferase leading to aberrant glycosylation of protein substrates. We therefore proceeded to assess for differences in enzymatic activities of respective wild-type versus each of the mutant versions of B3GNT2, B4GALT2, and ST6GALNAC2 transferases identified in this study (Table 2), using *in vitro* derived substrates (Supplementary Fig. S2).

**Figure 2:**
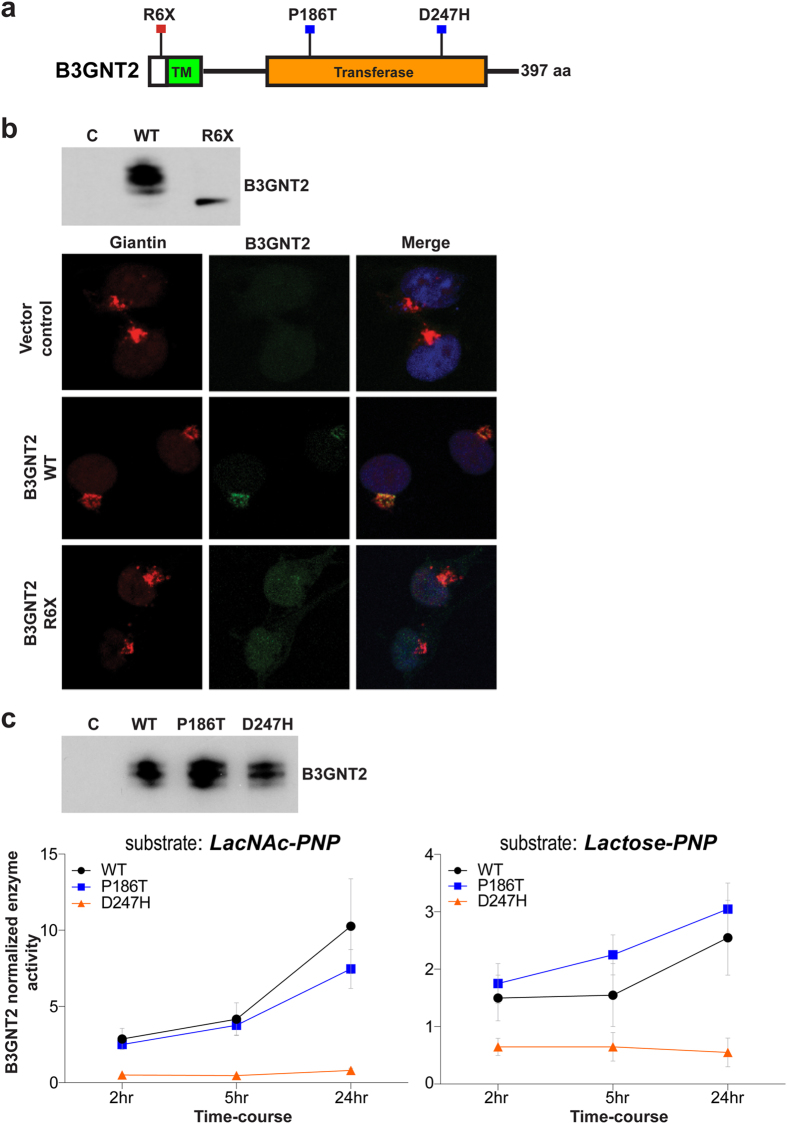
Biochemical characterization of wild-type and mutant versions of B3GNT2. (**a**) Somatic mutations mapped to B3GNT2 protein coding regions (black line). Colored boxes indicate annotated protein structural domains. (TM) transmembrane motif. (**b**) (top) Western blot analysis showing V5-tagged wild-type and R6X mutant protein expression in COS7 cells transfected with respective cDNA constructs. Note the relatively smaller size of mutant R6X protein as compared to wild-type B3GNT2. (**b**) (bottom) Immunofluorescence analysis of V5-tagged wild-type and R6X mutant protein in COS7 cells transfected with respective cDNA constructs. Note the co-localization of wild-type B3GNT2 (green) with the Golgi marker Giantin (red), and the aberrant sub-cellular localization of R6X mutant (green). (**c**) (top) Western blot analysis showing protein expression of V5-tagged wild-type and missense mutant versions of B3GNT2 in COS7 cells transfected with respective cDNA constructs. (**c**) (bottom) Mean enzyme activity of wild-type, P186T, and D247H mutants assessed using Lactose-PNP and LacNAc-PNP substrates as a function of reaction time normalized to vector control. Error bars represent standard error of the means derived from three independent replicate experiments. Note the marked loss of D247H mutant enzyme activity against both the substrates, when compared to wild-type B3GNT2 (P ≤ 0.05).

B3GNT2 (β-1,3-N-Acetylglucosaminyltransferase 2) catalyzes the addition of β-3 N-acetylglucosamine onto a terminal β-4 linked galactose residue forming extended polylactosamine (polyLacNAc) chains composed of repeats of N-acetyllactosamine (β-Gal (1–4) β-GlcNAc(1–3))_n_^15^. PolyLacNAc chains may be found on *N*- and *O*-linked glycans, the latter potentially attached to Core 1, Core 2 and Core 3 base structures as shown in Fig. 1^14^,^16^. As mentioned above, we identified 3 mutations in *B3GNT2*: R6X, P186T, and D247H (Table 2). Given the putative deleterious nature of the R6X stop-gain mutation (Fig. 2a), we first tested if this mutation leads to nonsense-mediated decay (NMD) of the transcript. RNA expression analysis of B3GNT2 in the corresponding mutant CRC cell line however showed retention of the mutant allele (Supplementary Fig. S3), suggesting this mutation may not activate NMD but may rather encode a truncated version of the protein via utilization of an alternative downstream translation start site. Western blot analysis of ectopically expressed R6X B3GNT2 into COS7 cells indeed showed a truncated protein product, albeit expressed at a significantly lower level than wild-type B3GNT2 protein (Fig. 2b). Mass spectrometry analysis further confirmed protein translation of R6X B3GNT2, but attempts to identify the start codon in the R6X mutant product were unsuccessful (data not shown). Nevertheless, given that the Golgi-targeting signal sequence is contained within the N-terminal B3GNT2 transmembrane motif (Fig. 2a), we hypothesized that the truncated R6X protein product may be devoid of the signal sequence and therefore would not localize to Golgi. Immunofluorescence analyses of ectopically expressed wild-type and R6X B3GNT2 in COS7 cells in fact showed wild-type B3GNT2 as being exclusively localized to the Golgi, in contrast to the R6X mutant which showed aberrant and diffuse sub-cellular localization (Fig. 2b). Taken together, these findings suggest that the miss-localized R6X B3GNT2 mutant may potentially lack access to endogenous substrates within the Golgi, or may aberrantly glycosylate unintended substrates within the cell.

The two missense B3GNT2 mutations (P186T and D247H) mapped to the catalytic domain of the B3GNT2 protein (Table 2, Fig. 2a, Supplementary Fig. S3). We assessed the impact of these mutations on encoded enzymatic activities using two different substrates, Lactose-PNP (Lactose para-nitrophenol) and LacNAc-PNP (LacNAc para-nitrophenol)^15^,^19^,^20^, selected based on the positive activity of wild-type B3GNT2 against each of these substrates (Supplementary Fig. S2). As shown in Fig. 2c, biochemical analysis revealed that while activities of the wild-type and P186T B3GNT2 proteins were comparable, the D247H mutant exhibited no detectable enzymatic activity against either of these substrates (P ≤ 0.05). Taken in total, these findings suggest that R6X and D247H mutations may markedly impair B3GNT2 downstream function in the cell.

**Figure 3:**
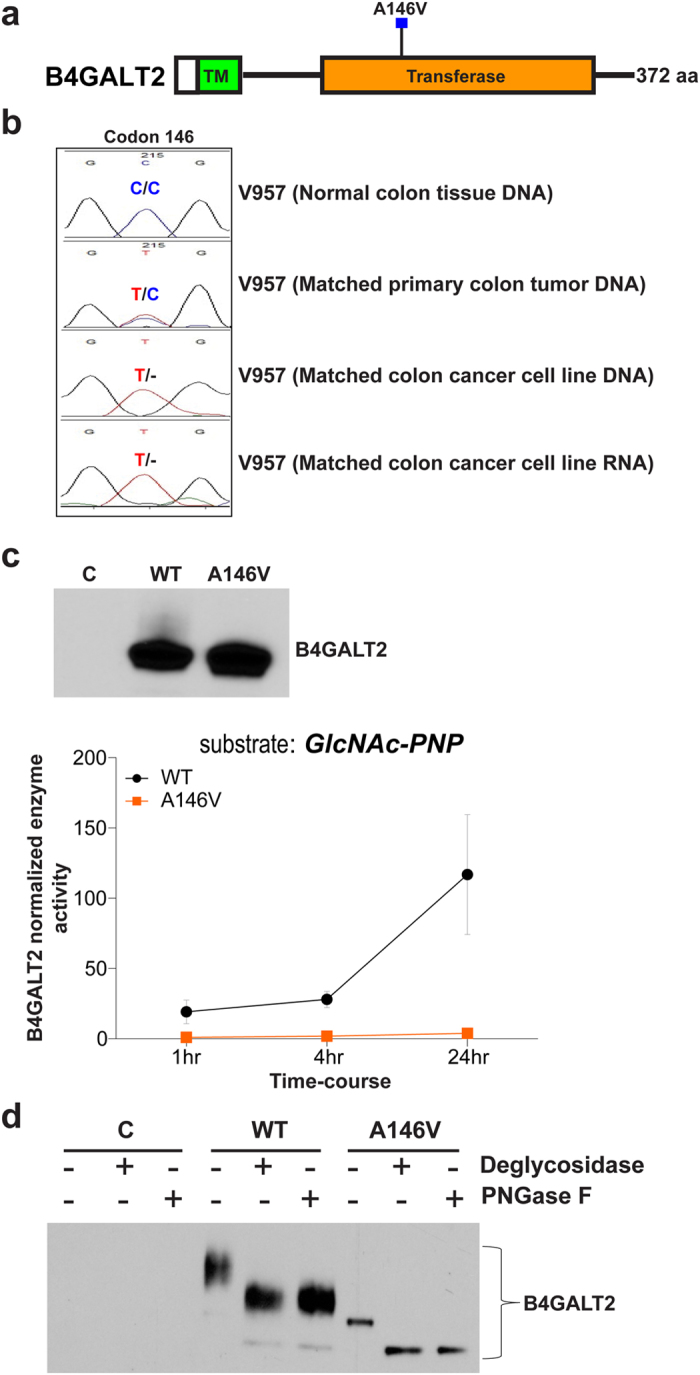
Biochemical characterization of wild-type and mutant B4GALT2. (**a**) A146V somatic mutation mapped to B4GALT2 protein coding regions (black line). Colored boxes indicate annotated protein structural domains. (TM) transmembrane motif. (**b**) Sequencing chromatograms depicting A146V somatic mutation in the matched primary tumor and cell line DNA, and cell line RNA. Note the loss of wild-type B4GALT2 allele in both the DNA and RNA from the cell line. (**c**) (top) Western blot analysis showing V5-tagged wild-type and mutant B4GALT2 protein expression in COS7 cells transfected with respective cDNA constructs. (**c**) (bottom) Mean enzyme activity of wild-type and mutant B4GALT2 proteins assessed using GlcNAc-PNP substrate as a function of incubation time, normalized to vector control. Error bars represent standard error of the means derived from three independent replicate experiments. Note the significant loss of A146V mutant enzyme activity when compared to wild-type protein (P ≤ 0.05). (**d**) Protein lysates from V957 cells transiently transfected with V5-tagged empty vector, wild-type or A146V B4GALT2 were immunoprecipitated with anti-V5 agarose and treated with either a pan-glycosidase, *N*-linked glycosidase PNGase F or left untreated. Western blot analysis was performed using anti-V5 antibody (see Methods). Note the significant difference in protein sizes between wild-type versus mutant B4GALT2 in untreated cells (lane 4 vs. 7 from left). Although both wild-type and mutant B4GALT2 proteins appear to be N-glycosylated (lanes 5, 6 vs. 8, 9 from left), a substantial fraction of glycosidase-treated wild-type protein still showed a higher size-shift than the mutant, suggesting wild-type B4GALT2 as selectively undergoing additional post-translational modifications.

B4GALT2 (β-1,4-Galactosyltransferase 2)catalyzes the transfer of galactose to N-acetylglucosamine residues forming the β-Gal(1–4) β-(GlcNAc)-R moiety on *N*- and *O*-linked glycans likely including the polyLacNAc structure (Fig. 1)^21^,^22^,^23^. The missense mutation, A146V, maps to the transferase catalytic domain of the *B4GALT2* gene (Fig. 3a). Interestingly, this mutation was accompanied by a genomic loss of the wild-type allele in the mutant CRC cell line (Fig. 3b). We next examined the impact of A146V mutation on the encoded B4GALT2 enzyme activity using a glucopyranoside substrate^21^, selected based on positive activity of wild-type B4GALT2 against this substrate (Supplementary Fig. S2). As shown in Fig. 3c, biochemical analysis revealed robust enzyme activity of the wild-type protein, with the A146V exhibiting no detectable enzymatic activity (P ≤ 0.05).

Interestingly, we also consistently noted that the wild-type B4GALT2 but not the A146V mutant as exhibiting a differential migratory pattern on SDS-PAGE, suggesting a potential post-translational modification of the wild-type protein (Fig. 3c). To test this, we ectopically expressed wild-type or A146V into the corresponding B4GALT2-mutant V957 CRC cell line, and performed Western blot analyses. Similar to our observations in COS7 cells (Fig. 3c), wild-type B4GALT2 protein exhibited a differential migratory pattern than the A146V mutant in V957 (Fig. 3d, lane 4 vs. 7 from left). Mass spectrometry analysis of respective protein bands in the wild-type and A146V mutant transfections confirmed their identity as B4GALT2 protein (data not shown), but was however unable to resolve the specific post-translation modification of wild-type B4GALT2. Nonetheless, given that B4GALT2 contains three potential *N*-linked glycosylation sites (NXS/T) at amino acids 66, 71, and 357^24^, we treated V957 cells ectopically expressing wild-type or A146V mutant proteins with either a pan *N*- and *O*-glycosidase or a specific *N*-linked glycosidase (PNGase F) to assess for *N*-linked glycosylation of wild-type versus mutant protein. While Western blot analysis showed both wild-type and mutant proteins as being predominately *N*-glycosylated (i.e. similar shifts with the pan-glycosidase and PNGase F), the wild-type protein still showed a higher size-shift than the mutant suggesting additional, as yet undetermined, post-translational modification of wild-type B4GALT2 (Fig. 3d, lanes 5, 6 vs. 8, 9 from left). These findings, besides revealing bi-allelic defects in B4GALT2, also suggest that mutational changes in B4GALT2 may potentially disrupt post-translational modification of the encoded protein, resulting in impaired enzymatic activity.

**Figure 4:**
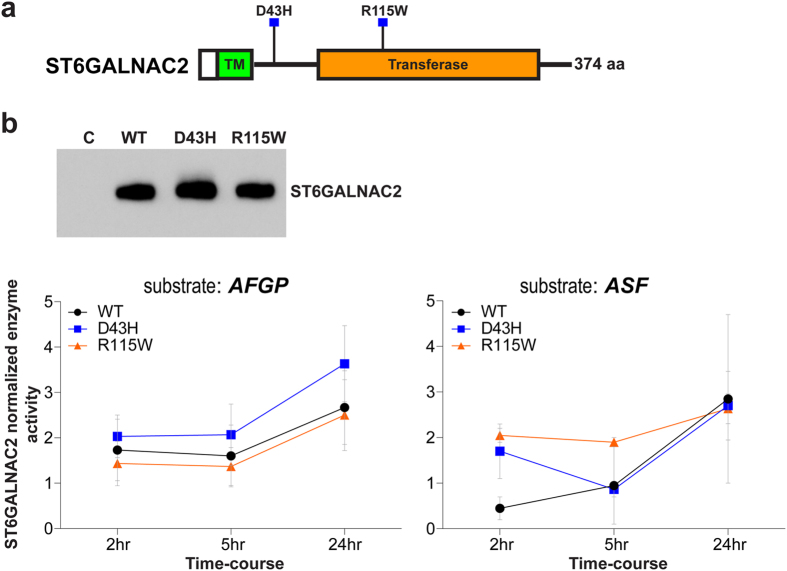
Biochemical characterization of wild-type and mutant versions of ST6GALNAC2. (**a**) Somatic mutations mapped to ST6GALNAC2 protein coding regions (black line). Colored boxes indicate annotated protein structural domains. (TM) transmembrane motif. (**b**) (top) Western blot analysis showing protein expression of V5-tagged wild-type and missense mutant versions of ST6GALNAC2 in COS7 cells transfected with respective cDNA constructs. (**b**) (bottom) Mean enzyme activity of wild-type and mutant ST6GALNAC2 protein assessed using antifreeze glycoprotein (AFGP) and asialofetuin (ASF) substrates at the indicated time points normalized to vector control. Error bars represent standard error of the means derived from three independent replicate experiments.

ST6GALNAC2 (α-N-Acetylgalactosaminidyl α-2,6-Sialyltransferase 2) catalyzes the addition of sialic acid residues to the 6 position of the peptide linked GalNAc in the Core 1 and Core 2 *O*-glycan structures: β-Gal(1–3) α-GalNAc-*O*-Thr/Ser and β-GlcNAc (1–3)α-GalNAc-*O*-Thr/Ser respectively (see Supplementary Fig. S2)^25^,^26^. We identified two missense mutations in the *ST6GALNAC2* gene, D43H located in the stalk between the transmembrane and transferase domain, and R115W located within the transferase domain (Fig. 4a, Supplementary Fig. S4). We assessed the impact of these mutations on encoded ST6GALNAC2 enzyme activity using antifreeze glycoprotein from Antarctic fish (AFGP) and asialofetuin (ASF) substrates^26^. AFGP consists of the (β-Gal(1–3)α-GalNAc-*O*-Thr-Ala-Ala)_n_ repeat, while ASF contains multiple O-glycan structures including β-Gal(1–3)α-GalNAc-*O*-Thr/Ser)^26^,^27^, both of which display incorporation of radiolabeled NeuNAc when wild-type ST6GALNAC2 is expressed (Supplementary Fig. S2). As shown in Fig. 4b, no significant differences in enzyme activities between wild-type and mutant ST6GALNAC2 was observed against these substrates, although we consistently observed an increased enzyme activity of the D43H mutant over the wild-type transferase against the AFGP substrate in our assays, which we did not observe with the ASF substrate. The significance of this apparent gain of activity in the mutant against the homogeneous AFGP is yet to be determined.

### Phenotypic characterization of wild-type and mutant glycosyltransferases

**Figure 5:**
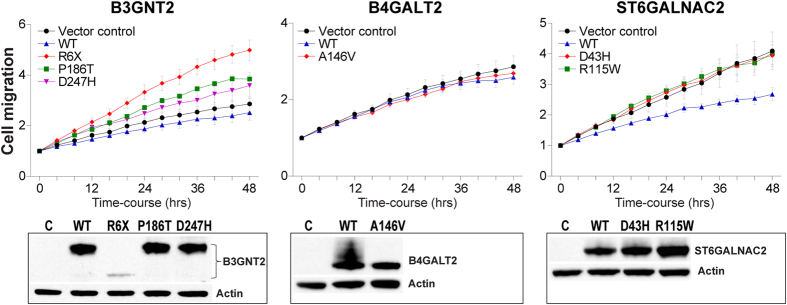
Effects of wild-type versus mutant enzymes on colon cancer cell migration. Migratory kinetics of SW480 CRC cells transiently expressing respective wild-type or mutant B3GNT2, ST6GALNAC2, B4GALT2 proteins were quantified using the IncuCyte scratch wound migration assay over a 48 hour time-course. Each of the mutant B3GNT2 expressing cells showed a significant increase in migratory potential when compared to the vector control (R6X, P = 0.001; P186T, P = 0.006, D247H, P = 0.047), whereas wild-type but not mutant ST6GALNAC2 significantly reduced cell migration as compared to vector control (P < 0.05) at the 48hr time-point. Error bars represent standard error of the means derived from five replicates per experimental group.

We next proceeded to examine the phenotypic consequences of the mutant glycosyltransferases identified in our CRC dataset (Table 2). Since aberrations in cell surface glycans have been shown to primarily affect the migratory and metastatic potential of cancer cells^28^,^29^,^30^,^31^, we compared the effects of wild-type versus mutant genes on cancer cell migration using the widely employed SW480 CRC cell line model. Of note, SW480 parental CRC cells show retention of endogenous B3GNT2 and B4GALT2 RNA expression with marked loss of expression of ST6GALNAC2 when compared to normal colon epithelia (Supplementary Fig. S5). SW480 cells were transiently transfected with respective wild-type or mutant versions of B3GNT2, ST6GALNAC2 and B4GALT2, or with an empty vector control, and cell migration was assessed in a scratch wound assay over a course of 48 hours using the highly quantitative IncuCyte live cell kinetic imaging system. While wild-type B3GNT2 showed no effect on cell migration, the mutant versions of B3GNT2 however significantly enhanced the migratory potential of SW480 cells (Fig. 5, P < 0.05), suggesting a potential gain of oncogenic function of the respective B3GNT2 mutant proteins. In contrast, wild-type ST6GALNAC2 markedly suppressed CRC cell migration (Fig. 5, P < 0.05), consistent with its proposed function as a tumor suppressor in breast cancer^32^, while the two ST6GALNAC2 mutants failed to inhibit cancer cell migration (Fig. 5) indicating potential loss of phenotypic function of the ST6GALNAC2 mutant proteins. No change in cell migration was observed in CRC cells carrying either wild-type or mutant B4GALT2 (Fig. 5). Taken together, these findings suggest that the endogenous protein targets of B3GNT2 and ST6GALNAC2 may likely be involved in regulating cell migration.

## Discussion

Aberrant protein glycosylation is a frequent pathological alteration associated with the onset and progression of colon cancers. Yet, the molecular mechanisms underlying aberrant glycosylation and their potential role in tumor progression remain poorly understood. Here, we performed targeted re-sequencing of 430 glycosylation-associated genes in 31 patient-derived CRC cell lines and matched primary colon tumors to characterize the type and extent of glycosylation pathway defects in colon cancer. Of the 430 genes tested, 12 genes were significantly mutated in CRCs (Table 1). In particular, we noticed an enrichment of mutations in the polylactosamine and *N-* and *O*-glycosylation pathway genes, including *B3GNT2*, *ST6GALNAC2*, and *B4GALT2* in CRCs (Table 1, Fig. 1). Together, mutations in these genes were detected in 5 of the 31 CRC cases tested, with 3 mutations in *B3GNT2* (R6X, P186T, D247H), 2 mutations in *ST6GALNAC2* (D43H, R115W), and 1 mutation in *B4GALT*2 (A146V) accompanied by a loss of the wild-type allele (Table 2, Fig. 3b). Additional evaluation of independent large-scale cancer datasets^17^,^18^ revealed recurrent somatic mutations in *B3GNT2*, *B4GALT2* and *ST6GALNAC2*, accounting for ~3% of CRC cases (Supplementary Table S6).

Functionally, B3GNT2 and to a lesser extent B4GALT2 are involved in the synthesis of polyLacNAc chains on *N*-linked tetraantennary structures and on Core 1, 2 and 3 *O*-glycan core structures (Fig. 1)^15^,^16^,^20^,^21^. ST6GALNAC2 on the other hand adds a NeuNAc to the 6 position of the peptide GalNAc of *O*-glycan Core 1 or 3 structures thus terminating chain elongation^26^,^33^. Although polyLacNAc biosynthesis and *O*-glycan core termination are independent processes they nevertheless may be linked, as the Core 1, 2 and 3 *O*-glycans may be further elongated with polyLacNAc chains (Fig. 1)^15^,^16^.

The *O*-glycosylation pathway is fundamental to several critical processes in the cell and aberrations in the *O*-glycosylation pathway are known to be associated with both early as well as later stages of cancer progression^3^,^9^,^34^. In particular, Core 3 *O*-glycans have been implicated in the maintenance of intestinal homeostasis^28^,^29^,^35^. In fact, Core 3 *O*-glycans are primarily expressed in gastrointestinal mucosa, and are the major core structures of mucin-type glycoproteins in colonic tissue^36^,^37^,^38^,^39^. Existing evidence also suggests that aberrations in Core 3 *O*-glycans likely play a key role in CRC development. For example, deregulated expression of Core 3 structures is frequently observed in colon cancers^40^,^41^. In particular, reduced expression of Core 3 synthase, an enzyme involved in the initial step of Core 3 biosynthesis, has been observed frequently in colon, gastric, and pancreatic ductal adenocarcinomas, with loss of Core 3 synthase expression highly correlating with the grade of colon neoplasia in familial adenomatous polyposis patients^28^,^29^. Furthermore, loss of activity of Core 3 synthase has been shown to enhance the metastatic potential of colon carcinoma cells^28^, and mice deficient in Core 3 synthase display reduced production of colonic MUC2 protein and show increased susceptibility to colitis and colon adenocarcinoma^42^,^43^. These findings, together with our observation of a significant enrichment of CRC-associated mutations in genes likely involved in Core 3 termination or Core 3 polyLacNAc elongation (Table 1, Fig. 1), strongly suggest that aberrations in these glycosyltransferases play an important role in CRC progression. We therefore proceeded to systematically characterize the functional consequences of each of the CRC-associated mutant glycosyltransferases identified in this study (Table 2).

As mentioned above, we detected 3 somatic mutations in *B3GNT2* (R6X, P186T, D247H). The R6X mutation, despite being a nonsense variant, encoded an N-terminal truncated protein (Fig. 2, Supplementary Fig. S3). Importantly, as opposed to the Golgi-specific localization of wild-type B3GNT2, the R6X mutant exhibited aberrant and diffuse sub-cellular localization (Fig. 2), suggesting that the mis-localized R6X mutant may be unable to access its endogenous substrates in the Golgi besides also potentially altering the glycosylation patterns of unintended substrates within the cell. Next, biochemical analyses of the missense mutants (P186T, D247H) using two *in vitro* derived B3GNT2 substrates (LacNAc-PNP and Lactose-PNP) showed a loss of enzymatic activity of the D247H mutant against these selected substrates (Fig. 2). Intriguingly, phenotypic analyses showed all three B3GNT2 mutants as significantly enhancing the migratory potential of colon adenocarcinoma cells (Fig. 5), indicating a gain of oncogenic function likely resulting from dominant negative activities of the mutant enzymes against wild-type B3GNT2 and/or other glycosyltransferases. Moreover, since B3GNT2 is involved in the synthesis of polyLacNAc chains, genetic defects in B3GNT2 could lead to aberrations in cell surface polylactosamines, critical signaling molecules that are often implicated in tumor cell migration and possibly metastasis^41^,^42^,^43^,^44^,^45^,^46^. Further studies to identify the actual endogenous substrates of B3GNT2 would help delineate the role of this transferase in the pathogenesis of CRCs.

The missense mutation (A146V) in B4GALT2 was accompanied by a genomic loss of the wild-type allele in the corresponding CRC sample (Fig. 3). Biochemical analyses using an *in vitro* derived, B4GALT2 substrate, GlcNAc-PNP, showed loss of enzymatic activity of the A146V mutant (Fig. 3). In addition, de-glycosylation studies revealed A146V mutation as markedly affecting post-translational modification of the B4GALT2 protein (Fig. 3), which in turn could have a negative impact on its enzymatic activity. Phenotypic analysis however showed neither wild-type nor mutant B4GALT2 as affecting the migratory potential of CRC cells (Fig. 5), suggesting that the endogenous targets of B4GALT2 may not likely be involved in regulating cell motility or that they require tissue microenvironment for functioning.

The two missense mutations detected in *ST6GALNAC2* (D43H and R115W) showed no apparent loss of enzymatic activities when tested against the AFGP and ASF substrates, although the D43H mutant consistently showed an increase in enzyme activity over the wild-type transferase against the AFGP substrate in our assays (Fig. 4). This finding of enhanced D43H enzyme activity appears to be in keeping with prior studies where sialylation is increased while Core 3 structures decreased in CRC^47^. Interestingly, phenotypic analyses demonstrated wild-type ST6GALNAC2, but not the mutants, as markedly impeding the migratory potential of colon carcinoma cells (Fig. 5). It is likely that the wild-type and mutant ST6GALNAC2 proteins may exhibit differential specificities/affinities towards actual endogenous protein targets involved in regulating cell migration *in vivo*. Nonetheless, our phenotypic findings are consistent with the reported metastasis suppressor role of ST6GALNAC2 in breast cancer^32^ and further indicate a loss of phenotypic function of ST6GALNAC2 mutants identified in CRCs.

In summary, we have comprehensively characterized the mutational landscapes of glycosylation-associated genes in colon cancer, identifying three glycosyltransferases as significant mutational targets in CRCs. Functional studies demonstrate these mutant glycosyltransferases as having a significant impact on the encoded enzymatic activity and/or the migratory potential of colon carcinoma cells. Although our study may not fully capture the functional complexities and kinetics of *N*- or *O*-linked glycosylation, the finding of functionally deleterious CRC mutations in genes that are likely fundamental to maintaining intestinal homeostasis, suggests that genetic defects in polylactosamine and Cores 1 and 3 *O*-glycosylation pathway potentially contribute to CRC pathogenesis. Of note, given our prior studies identifying mutations in *GALNT12* gene as being associated with susceptibility to familial colon neoplasia^9^,^48^, future studies can be designed to explore whether genetic defects in Core 1/3 glycosylation pathway also play a role in susceptibility to unexplained inherited forms of colon cancer. Further characterization of the actual endogenous substrates of these glycosyltransferases and evaluation of phenotypic consequences of these mutant glycosyltransferases in pre-clinical animal models should provide additional insights into the biologic role of these genes in colon cancer progression.

## Materials and Methods

Detailed methods are provided in Supplementary Methods section.

### Patient samples and nucleic acid extraction

Patient-derived VACO series of colon cancer cell lines were propagated as previously describedz. Colon tumor and normal tissue specimens matched to respective VACO cell lines were obtained from a formalin-fixed paraffin embedded (FFPE) archive that were collected under an Institutional Review Board (IRB) approved protocol at the Case Medical Center. All participants provided written informed consent prior to participating in the study and all methods were carried out in accordance with the approved guidelines. Genomic DNA from the cell lines and FFPE tissues was extracted as previously described^49^,^50^. Demographics of DNA samples used for the study are provided in Supplementary Table S1.

### Targeted re-sequencing of glycome pathway genes

A custom Agilent SureSelect XT array (Agilent Technologies, Inc. Santa Clara, CA) was designed to capture and sequence the coding and splice site regions of 430 candidate glycosylation pathway genes (Supplementary Table S2) in a series of 31 patient-derived VACO CRC cell lines (Supplementary Table S1).

### Somatic mutation detection

Burrows-Wheeler Aligner^51^ was used to align the raw FASTQ files to the human reference genome (build hg19). Sample coverage metrics are provided in Supplementary Table S3. Nucleotide variations were detected using SOAPsnp^52^, Genome Analysis Toolkit^53^ and mPILEUP^54^. Somatic mutations were identified using a series of variant-filtering steps, and were confirmed by Sanger sequencing in both cell lines and matched primary colon tumor tissues. All together, 41 somatic protein-altering mutations in 36 genes were identified amongst the 31 CRC cases tested (Supplementary Table S4).

### Significantly mutated genes and selection of gene candidates for functional studies

Significantly mutated genes were identified using the statistical framework previously described by our group^12^. Twelve candidate genes showed a significantly higher mutation rate than the background (Supplementary Table S5). Three of these were identified as genes involved in the polylactosamine chain extension on *N*- and Core 1–3 *O*-linked glycans or in chain termination of Core 1/3 *O*-glycans, and were selected for further functional characterization (Supplementary Table S5).

### Sanger sequencing

Custom PCR primers flanking respective mutant loci in candidate glycosyltransferase genes were designed for Sanger sequencing (Supplementary Table S7).

### Pyrosequencing to test for *KRAS/BRAF* hotspot mutations

Pyrosequencing assays were designed using the PSQ Assay Design software (QIAGEN, Chatsworth, CA) to test for hotspot mutations in KRAS (codons 12, 13, 61, and 146) and BRAF (codon 600). For each assay, one of the PCR primers was biotinylated at the 5′ end and purified using high performance liquid chromatography. All PCR reactions were performed using FastStart Taq (Roche). Following PCR, amplification products were sequenced on a PyroMark MD pyrosequencing instrument (QIAGEN) and mutation analysis was conducted as previously described^50^. Sanger sequencing was used to confirm all mutations detected by pyrosequencing analysis.

### Mutual Exclusivity Evaluation

To test if mutations affecting the glycosylation genes occur in a mutually exclusive fashion with respect to other known oncogenic driver mutations in CRCs (KRAS and BRAF), we applied CoMEt^55^, which employs an exact statistical test for mutual exclusivity that has been shown to be more sensitive in detecting mutually exclusive events within combinations containing rare alterations.

### Generation of expression constructs and recombinant protein purification

Full length cDNA fragments, encoding wild-type (WT) or mutant B3GNT2, ST6GALNAC2 and B4GALT2 transcripts were PCR amplified from total RNA derived from a reference normal colon sample or from corresponding mutant CRC cell lines, respectively, and cloned into *pcDNA3.1* or *pIHV* vectors. Transfection was performed in COS7 cells using Lipofectamine 2000 (Life technologies, Carlsbad, CA); recombinant proteins were isolated using immunoprecipitation with anti-V5 antibody.

### Western blot analysis

1/10^th^ of the immunoprecipitated recombinant protein was subjected to SDS/PAGE analysis and immunoblotted with mouse anti-V5 antibody.

### Enzyme assays

The donor and acceptor substrates used for assaying wild-type B3GNT2, B4GALT2, and ST6GALNAC2 enzyme activities are given in Supplementary Fig. S2A. Briefly, reaction mix containing 100–150 μl of immunoprecipitated proteins were subjected to either reverse-phase chromatography (B3GNT2, B4GALT2) or dialysis (ST6GALNAC2) to measure the incorporation of radio-labelled sugars (UDP-[^3^H]-GlcNAc, UDP-[^3^H]-Gal, CMP-[^3^H]-NeuNAc) by respective wild-type and mutant enzymes over a period of 24 hrs.

### Mass spectrophotometry

COS7 cells transfected with empty vector, wild-type or mutant expression constructs of B3GNT2 or B4GALT2 were immunoprecipitated with anti-V5 antibody and subjected to SDS-PAGE. Relevant Coomassie G250 stained protein bands were excised for subsequent Mass spectrometry analysis using liquid chromatography-tandem mass spectrometry (LC-MS/MS).

### *In vitro* glycosidase assay

V957 CRC cells were transfected with *pcDNA3.1*/V5-His/empty vector, or B4GALT2 wild-type or B4GALT2 A146V mutant constructs. Immunoprecipitated wild-type and mutant B4GALT2 protein were treated with either Peptide N-glycosidase F or a pan glycosidase protein deglycosylation mix or left untreated at 37 °C for 4 hours followed by Western blot analysis using anti-V5 antibody.

### Confocal Imaging

COS7 cells transfected with V5-tagged *pcDNA3.1* empty vector or B3GNT2 wild-type or B3GNT2 R6X mutant were immunostained with anti-V5 antibody, anti-Giantin antibody and DRAQ5 (nuclear counterstain). Immunostained cells were visualized using the Zeiss LSM 510 confocal microscope.

### Scratch wound cell migration assay

Scratch wound assay was performed in SW480 cells, transfected with either *pcDNA3.1/pIHV* empty vector, respective wild-type or mutant constructs of B3GNT2, B4GALT2, and ST6GALNAC2, using the automated IncuCyte ZOOM live cell kinetic imaging system (Essen BioScience, Ann Arbor, MI) as per the manufacturer’s instructions over a period of 48hrs.

### Statistical analyses

Significant differences in enzyme activities and cell migration between wild-type and mutant proteins were estimated using a Student’s t-test; a P value < 0.05 was considered statistically significant.

## Additional Information

**How to cite this article**: Venkitachalam, S. *et al.* Biochemical and functional characterization of glycosylation-associated mutational landscapes in colon cancer. *Sci. Rep.* **6**, 23642; doi: 10.1038/srep23642 (2016).

## Supplementary Material

Supplementary Information

Supplementary Datset

## Figures and Tables

**Table 1:** Functional annotation of glycosylation pathway genes found significantly mutated in colon cancer.

Gene Symbol	Glycosylation Pathway sub-network
*B3GALNT1*	Globoside synthesis
*B3GALT1*	Glycosphingolipid synthesis, N-glycan trimming and branching
*B3GNT2*	*N-/Core 1,2,3, O-linked glycan biosynthesis, polylactosamine*
*B4GALT2*	*N-/Core 1,2,3, O-linked glycan biosynthesis, polylactosamine*
*CHST12*	Sulfation of Chondroitin and Dermatan
*CMAS*	Nucleotide sugar biosynthesis
*GAL3ST1*	Glycosphingolipid synthesis, Isogloboside synthesis
*GAL3ST3*	Isogloboside synthesis
*GLB1*	Hydrolysis of Beta-galactose
*HS3ST5*	Sulfation of Heparan
*LYZ*	Lysozyme activity
*ST6GALNAC2*	*Core 1,3, O-linked glycan biosynthesis*

**Table 2:** Colon cancer associated somatic mutations in *B3GNT2, ST6GALNAC2* and *B4GALT2* genes.

Gene Symbol	CRC Sample I.D	Variant class	Protein change	UniProt Domain	SIFT/Polyphen Prediction	Loss of wild-type allele in cancer DNA/RNA
*B3GNT2*	V964	Nonsense	R6X	Topological	N/A	No
*B3GNT2*	V915	Missense	P186T	Transferase	Deleterious	No
*B3GNT2*	V920	Missense	D247H	Transferase	Deleterious	No
*ST6GALNAC2*	V915	Missense	D43H	Stalk	Deleterious	No
*ST6GALNAC2*	V451	Missense	R115W	Transferase	Neutral	No
*B4GALT2*	V957	Missense	A146V	Transferase	Deleterious	Yes

## References

[b1] Ohtsubo K. & Marth J. D. Glycosylation in cellular mechanisms of health and disease. Cell 126, 855–867 (2006). 16959566 10.1016/j.cell.2006.08.019

[b2] Stanley P. Golgi glycosylation. Cold Spring Harb Perspect Biol 3, doi: 10.1101/cshperspect.a005199 (2011). PMC3062213 21441588

[b3] Pinho S. S. & Reis C. A. Glycosylation in cancer: mechanisms and clinical implications. Nat Rev Cancer 15, 540–555 (2015). 26289314 10.1038/nrc3982

[b4] Gill D. J., Clausen H. & Bard F. Location, location, location: new insights into O-GalNAc protein glycosylation. Trends Cell Biol 21, 149–158 (2011). 21145746 10.1016/j.tcb.2010.11.004

[b5] Kudelka M. R., Ju T., Heimburg-Molinaro J. & Cummings R. D. Simple sugars to complex disease–mucin-type O-glycans in cancer. Adv Cancer Res 126, 53–135 (2015). 25727146 10.1016/bs.acr.2014.11.002 PMC5812724

[b6] Brockhausen I., Schachter H. & Stanley P. In Essentials of Glycobiology (eds Varki A. *et al.*) (2009).

[b7] Bergstrom K. S. & Xia L. Mucin-type O-glycans and their roles in intestinal homeostasis. Glycobiology 23, 1026–1037 (2013). 23752712 10.1093/glycob/cwt045 PMC3858029

[b8] Tran D. T. & Ten Hagen K. G. Mucin-type O-glycosylation during development. J Biol Chem 288, 6921–6929 (2013). 23329828 10.1074/jbc.R112.418558 PMC3591602

[b9] Guda K. *et al.* Inactivating germ-line and somatic mutations in polypeptide N-acetylgalactosaminyltransferase 12 in human colon cancers. Proc Natl Acad Sci USA 106, 12921–12925 (2009). 19617566 10.1073/pnas.0901454106 PMC2722285

[b10] Kumar P., Henikoff S. & Ng P. C. Predicting the effects of coding non-synonymous variants on protein function using the SIFT algorithm. Nat Protoc 4, 1073–1081 (2009). 19561590 10.1038/nprot.2009.86

[b11] Sunyaev S. *et al.* Prediction of deleterious human alleles. Hum Mol Genet 10, 591–597 (2001). 11230178 10.1093/hmg/10.6.591

[b12] Sjoblom T. *et al.* The consensus coding sequences of human breast and colorectal cancers. Science 314, 268–274 (2006). 16959974 10.1126/science.1133427

[b13] Guda K. *et al.* Novel recurrently mutated genes in African American colon cancers. Proc Natl Acad Sci USA 112, 1149–1154 (2015). 25583493 10.1073/pnas.1417064112 PMC4313860

[b14] Mitsui Y. *et al.* Comparative studies on glycoproteins expressing polylactosamine-type N-glycans in cancer cells. J Pharm Biomed Anal 70, 718–726 (2012). 22795310 10.1016/j.jpba.2012.06.035

[b15] Togayachi A. *et al.* Beta3GnT2 (B3GNT2), a major polylactosamine synthase: analysis of B3GNT2-deficient mice. Methods Enzymol 479, 185–204 (2010). 20816167 10.1016/S0076-6879(10)79011-X

[b16] Nairn A. V. *et al.* Regulation of glycan structures in animal tissues: transcript profiling of glycan-related genes. J Biol Chem 283, 17298–17313 (2008). 18411279 10.1074/jbc.M801964200 PMC2427342

[b17] Cerami E. *et al.* The cBio cancer genomics portal: an open platform for exploring multidimensional cancer genomics data. Cancer Discov 2, 401–404 (2012). 22588877 10.1158/2159-8290.CD-12-0095 PMC3956037

[b18] Gao J. *et al.* Integrative analysis of complex cancer genomics and clinical profiles using the cBioPortal. Sci Signal 6, pl1 (2013). 10.1126/scisignal.2004088 PMC4160307 23550210

[b19] Shiraishi N. *et al.* Identification and characterization of three novel beta 1,3-N-acetylglucosaminyltransferases structurally related to the beta 1,3-galactosyltransferase family. J Biol Chem 276, 3498–3507 (2001). 11042166 10.1074/jbc.M004800200

[b20] Togayachi A., Sato T. & Narimatsu H. Comprehensive enzymatic characterization of glycosyltransferases with a beta3GT or beta4GT motif. Methods Enzymol 416, 91–102 (2006). 17113861 10.1016/S0076-6879(06)16006-1

[b21] Almeida R. *et al.* A family of human beta4-galactosyltransferases. Cloning and expression of two novel UDP-galactose:beta-n-acetylglucosamine beta1, 4-galactosyltransferases, beta4Gal-T2 and beta4Gal-T3. J Biol Chem 272, 31979–31991 (1997). 9405390 10.1074/jbc.272.51.31979

[b22] Nabi I. R. & Dennis J. W. The extent of polylactosamine glycosylation of MDCK LAMP-2 is determined by its Golgi residence time. Glycobiology 8, 947–953 (1998). 9675228 10.1093/glycob/8.9.947

[b23] Zhou J. *et al.* Identification of beta1,4GalT II as a target gene of p53-mediated HeLa cell apoptosis. J Biochem 143, 547–554 (2008). 18211920 10.1093/jb/mvn003

[b24] UniProt C. UniProt: a hub for protein information. Nucleic Acids Res 43, D204–212 (2015). 25348405 10.1093/nar/gku989 PMC4384041

[b25] Dall’Olio F., Malagolini N., Trinchera M. & Chiricolo M. Sialosignaling: sialyltransferases as engines of self-fueling loops in cancer progression. Biochim Biophys Acta 1840, 2752–2764 (2014). 24949982 10.1016/j.bbagen.2014.06.006

[b26] Kono M. *et al.* Redefined substrate specificity of ST6GalNAc II: a second candidate sialyl-Tn synthase. Biochem Biophys Res Commun 272, 94–97 (2000). 10872809 10.1006/bbrc.2000.2745

[b27] Berman E., Allerhand A. & DeVries A. L. Natural abundance carbon 13 nuclear magnetic resonance spectroscopy of antifreeze glycoproteins. J Biol Chem 255, 4407–4410 (1980). 7372583

[b28] Iwai T. *et al.* Core 3 synthase is down-regulated in colon carcinoma and profoundly suppresses the metastatic potential of carcinoma cells. Proc Natl Acad Sci USA 102, 4572–4577 (2005). 15755813 10.1073/pnas.0407983102 PMC555466

[b29] Radhakrishnan P. *et al.* Expression of core 3 synthase in human pancreatic cancer cells suppresses tumor growth and metastasis. Int J Cancer 133, 2824–2833 (2013). 23754791 10.1002/ijc.28322 PMC3873636

[b30] Seales E. C. *et al.* Hypersialylation of beta1 integrins, observed in colon adenocarcinoma, may contribute to cancer progression by up-regulating cell motility. Cancer Res 65, 4645–4652 (2005). 15930282 10.1158/0008-5472.CAN-04-3117

[b31] Hauselmann I. & Borsig L. Altered tumor-cell glycosylation promotes metastasis. Front Oncol 4, 28 (2014). 24592356 10.3389/fonc.2014.00028 PMC3923139

[b32] Murugaesu N. *et al.* An *in vivo* functional screen identifies ST6GalNAc2 sialyltransferase as a breast cancer metastasis suppressor. Cancer Discov 4, 304–317 (2014). 24520024 10.1158/2159-8290.CD-13-0287

[b33] Brockhausen I. Pathways of O-glycan biosynthesis in cancer cells. Biochim Biophys Acta 1473, 67–95 (1999). 10580130 10.1016/s0304-4165(99)00170-1

[b34] Stowell S. R., Ju T. & Cummings R. D. Protein glycosylation in cancer. Annu Rev Pathol 10, 473–510 (2015). 25621663 10.1146/annurev-pathol-012414-040438 PMC4396820

[b35] Kawashima H. Roles of the gel-forming MUC2 mucin and its O-glycosylation in the protection against colitis and colorectal cancer. Biol Pharm Bull 35, 1637–1641 (2012). 23037153 10.1248/bpb.b12-00412

[b36] Capon C., Maes E., Michalski J. C., Leffler H. & Kim Y. S. Sd(a)-antigen-like structures carried on core 3 are prominent features of glycans from the mucin of normal human descending colon. Biochem J 358, 657–664 (2001). 11577689 10.1042/bj3580657 PMC1222115

[b37] Podolsky D. K. Oligosaccharide structures of human colonic mucin. J Biol Chem 260, 8262–8271 (1985). 4008490

[b38] Podolsky D. K. Oligosaccharide structures of isolated human colonic mucin species. J Biol Chem 260, 15510–15515 (1985). 4066681

[b39] Brockhausen I., Matta K. L., Orr J. & Schachter H. Mucin synthesis. UDP-GlcNAc:GalNAc-R beta 3-N-acetylglucosaminyltransferase and UDP-GlcNAc:GlcNAc beta 1–3GalNAc-R (GlcNAc to GalNAc) beta 6-N-acetylglucosaminyltransferase from pig and rat colon mucosa. Biochemistry 24, 1866–1874 (1985). 3160388 10.1021/bi00329a010

[b40] Vavasseur F. *et al.* O-glycan biosynthesis in human colorectal adenoma cells during progression to cancer. Eur J Biochem 222, 415–424 (1994). 8020479 10.1111/j.1432-1033.1994.tb18880.x

[b41] Vavasseur F., Yang J. M., Dole K., Paulsen H. & Brockhausen I. Synthesis of O-glycan core 3: characterization of UDP-GlcNAc: GalNAc-R beta 3-N-acetyl-glucosaminyltransferase activity from colonic mucosal tissues and lack of the activity in human cancer cell lines. Glycobiology 5, 351–357 (1995). 7655172 10.1093/glycob/5.3.351

[b42] An G. *et al.* Increased susceptibility to colitis and colorectal tumors in mice lacking core 3-derived O-glycans. J Exp Med 204, 1417–1429 (2007). 17517967 10.1084/jem.20061929 PMC2118614

[b43] Xia L. Core 3-derived O-glycans are essential for intestinal mucus barrier function. Methods Enzymol 479, 123–141 (2010). 20816163 10.1016/S0076-6879(10)79007-8

[b44] Togayachi A. *et al.* Polylactosamine on glycoproteins influences basal levels of lymphocyte and macrophage activation. Proc Natl Acad Sci USA 104, 15829–15834 (2007). 17890318 10.1073/pnas.0707426104 PMC2000437

[b45] Kinoshita M. *et al.* Common glycoproteins expressing polylactosamine-type glycans on matched patient primary and metastatic melanoma cells show different glycan profiles. J Proteome Res 13, 1021–1033 (2014). 24354860 10.1021/pr401015b

[b46] Ni J. *et al.* beta3GnT8 regulates the metastatic potential of colorectal carcinoma cells by altering the glycosylation of CD147. Oncol Rep 31, 1795–1801 (2014). 24573103 10.3892/or.2014.3042

[b47] Holst S., Wuhrer M. & Rombouts Y. Glycosylation characteristics of colorectal cancer. Adv Cancer Res 126, 203–256 (2015). 25727149 10.1016/bs.acr.2014.11.004

[b48] Clarke E. *et al.* Inherited deleterious variants in GALNT12 are associated with CRC susceptibility. Hum Mutat 33, 1056–1058 (2012). 22461326 10.1002/humu.22088

[b49] Adams M. D. *et al.* Global mutational profiling of formalin-fixed human colon cancers from a pathology archive. Mod Pathol 25, 1599–1608 (2012). 22878650 10.1038/modpathol.2012.121 PMC3697090

[b50] Fecteau R. E., Lutterbaugh J., Markowitz S. D., Willis J. & Guda K. GNAS mutations identify a set of right-sided, RAS mutant, villous colon cancers. PLoS One 9, e87966 (2014). 24498230 10.1371/journal.pone.0087966 PMC3907576

[b51] Li H. & Durbin R. Fast and accurate short read alignment with Burrows-Wheeler transform. Bioinformatics 25, 1754–1760 (2009). 19451168 10.1093/bioinformatics/btp324 PMC2705234

[b52] Li R. *et al.* SNP detection for massively parallel whole-genome resequencing. Genome Res 19, 1124–1132 (2009). 19420381 10.1101/gr.088013.108 PMC2694485

[b53] McKenna A. *et al.* The Genome Analysis Toolkit: a MapReduce framework for analyzing next-generation DNA sequencing data. Genome Res 20, 1297–1303 (2010). 20644199 10.1101/gr.107524.110 PMC2928508

[b54] Li H. *et al.* The Sequence Alignment/Map format and SAMtools. Bioinformatics 25, 2078–2079 (2009). 19505943 10.1093/bioinformatics/btp352 PMC2723002

[b55] Leiserson M. D., Wu H. T., Vandin F. & Raphael B. J. CoMEt: a statistical approach to identify combinations of mutually exclusive alterations in cancer. Genome Biol 16, 160 (2015). 26253137 10.1186/s13059-015-0700-7 PMC4531541

