# Supplementary material for: Biochemical and functional characterization of glycosylation-associated mutational landscapes in colon cancer
Source: Sci Rep. 2016 Mar 23;6:23642. doi: 10.1038/srep23642 (PMC4804330; doi:10.1038/srep23642)
Supplement: Supplementary Information [file srep23642-s1.doc]

**Supplementary Methods and Figures**

**Biochemical and functional characterization of glycosylation-associated mutational landscapes in colon cancer**

Srividya Venkitachalam, Leslie Revoredo, Vinay Varadan, Ryan Fecteau, Lakshmeswari Ravi, James Lutterbaugh, Sanford D. Markowitz, Joseph E. Willis, Thomas A. Gerken and Kishore Guda

**SUPPLEMENTARY METHODS**

**CRC cell lines, patient samples, and nucleic acid extraction**

The patient-derived VACO series of colon cancer cell lines were propagated as previously described ^1,2^. Colon tumor and normal tissue specimens matched to respective VACO cell lines were obtained from a formalin-fixed paraffin embedded (FFPE) archive that were collected under an Institutional Review Board (IRB) approved protocol at the Case Medical Center. Genomic DNA from the cell lines and FFPE tissues was extracted as previously described ^3,4^. DNA from the CRC cell lines was tested for microsatellite instability at microsatellite markers: BAT26, BAT40, D2S123, D5S346, and D17S250 ^5^. Only microsatellite stable (MSS) cell lines were included in this study. Total RNA from the cell lines was extracted using the mirVana Kit (Ambion, Austin, TX). The tumor stage, gender, race, MSI status, and tissue source of DNA samples used for the study are provided in Supplementary Table S1.

**Targeted re-sequencing of glycome pathway genes**

A total of 430 candidate genes, belonging to known protein glycosylation pathways, were identified (Supplementary Table S2) by reviewing published literature ^6-8^ and annotated databases, including the Consortium for Functional Glycomics (<http://www.functionalglycomics.org/fg/>). A custom Agilent SureSelect XT array was designed to capture and sequence the coding and splice site regions of the 430 genes (Agilent Technologies, Inc. Santa Clara, CA) in a series of 31 MSS VACO CRC cell lines (Supplementary Table S1). Sample library generation was carried out by the Gene Expression and Genotyping Core Facility at CWRU cancer center as per manufacturer’s instructions. Briefly, sample DNA’s were quantified using a picogreen fluorometric assay and 3µg of genomic DNA were randomly sheared to obtain a base pair peak of 150 bp using a Covaris S2 sonicator (**Covaris Inc,** Woburn, MA). Sonicated DNA was then end-repaired, A-tailed, and ligated with indexing-specific adapters. Each sample was captured using the custom capture array kit followed by library amplification and quality assessment using Agilent 2100 Bioanalyzer High Sensitivity DNA Chip (Agilent). The captured libraries were precisely quantified using a qPCR-based Kapa Biosystems library quantification kit (Kapa Biosystems Inc, Woburn, MA) on a Roche Lightcycler 480 (Roche Applied Science, Indianapolis, IN). Amplified libraries were pooled in batches of 10 or 11 samples per pool and sequenced using the Illumina HiScan instrument (Illumina Inc, San Diego, CA) to generate 100bp single-end reads.

**Read mapping and annotation**

Burrows-Wheeler Aligner (BWA) ^9^ was used to align individual 100bp reads from the raw FASTQ files to the human reference genome (build hg19) with default parameters. Following the conversion of aligned reads in to binary Sequence Alignment/Map (BAM) format, coverage metrics of target bases were calculated using the Picard algorithm ([http://samtools.sourceforge.net](http://samtools.sourceforge.net/)). Sample coverage metrics provided in Supplementary Table S3 show 94.31% of the target bases as being covered at 30X average read-depth, with 1.48% of target bases showing no coverage. Next, nucleotide variations (SNV, splice site, and insertion/deletion) in respective samples were detected using three variant calling algorithms including, SOAPsnp ^10^, Genome Analysis Toolkit (GATK) ^11^, and mPILEUP ^12^, set to default parameters, and with the human reference sequence as the background. Genomic variants were mapped to the human transcriptome reference database (RefSeq, build hg19) using a variant annotation tool developed in house (SLATE), which identifies variants mapping to gene coding regions and splice-sites, including their corresponding positions and codon changes within respective transcripts.

**Filtering of variants and detection of somatic mutations**

Since targeted re-sequencing was performed only on the CRC cell lines, a series of variant filtering steps detailed below were performed to identify putative somatic mutations. First, all the variants identified in the cell lines were queried against the 1000 genome database ^13^ as well as against an in-house variant database generated from platform-matched whole exome sequencing of more than 150 germline samples, to eliminate variants that are likely germline and not somatic. The use of platform-matched in-house database additionally aided in eliminating recurrent artifacts or false positives seen in the deep sequence data. Second, direct Sanger sequencing was performed on all the remaining putative somatic variants in cell lines, and any variants that were not confirmed by Sanger were eliminated. Third, each of the variants were Sanger sequenced in respective matched FFPE normal tissue to ascertain the somatic nature of the variants. Fourth, candidate somatic mutations were then Sanger sequenced in respective matched FFPE primary tumor tissue, and only mutations that were also present in the antecedent tumor tissue were included for further analysis. In the end, 41 somatic protein-altering mutations in 36 genes were identified amongst the 31 CRC cases (Supplementary Table S4).

**Significantly mutated genes and selection of gene candidates for functional studies**

In order to identify genes that were mutated at a significantly higher rate than the background, we followed a statistical framework similar to our published studies detailing mutational profiles of colon cancer ^14^. Briefly, to account for nucleotide composition influence on the likelihood of mutations, base coverage metrics for the six different nucleotide contexts including AT transitions, AT transversions, CG transitions, CG transversions, CpG transitions and CpG transversions were first obtained for each of the 36 genes (Supplementary Table S3) in the entire sample cohort using the Genome MuSiC suite ^15^. The background mutation rate for each of the above six mutation contexts and indels in colon cancers was derived from an in-house database of whole exome sequence data derived from 30 late-stage colon cancers (Supplementary Table S5). Next, for a given gene, we counted the number of mutations in each of the above seven categories (indels plus mutations in six different sequence contexts) and calculated the probability of the observed number of mutations in a particular category using an exact binomial distribution. The total probability (combined P value) of a gene exhibiting the observed number of mutations in all of the seven categories was then calculated to be the product of the seven context-specific probabilities. Finally, to correct these probabilities for multiple comparisons, we used the algorithm described by Benjamini and Hochberg ^16^. Overall, 12 of 36 candidate genes showed a significantly higher mutation rate than the background (P≤0.01, FDR <0.05) (Supplementary Table S5). 3 of these were identified as genes involved in the polylactosamine chain extension on *N*- and Core 1-3 *O*-linked glycans or in chain termination of Core 1/3 *O*-glycans, and were selected for further functional characterization (Supplementary Table S5).

**Sanger sequencing**

For DNA, gene-specific M13-tagged PCR primers flanking respective mutant loci were custom designed (Supplementary Table S7). PCR conditions included 95ºC for 4 min, 35 cycles of 95ºC for 45s, 62.3ºC for 30s and 72ºC for 45s. Each reaction was carried out in a 50µl reaction volume using 2U of Fast-TAQ DNA polymerase (Roche Applied Science, Indianapolis, IN) with 10-20ng of cell line, primary tumor or matched normal DNA. The PCR products were purified, and sequenced directly or sequenced following sub-cloning into *pCR2.1*-TOPO vector using universal M13 forward and reverse primers. For RNA, 2 µg DNase-treated RNA was reverse transcribed using SuperScript III First-Strand Synthesis (Life technologies, Carlsbad, CA) according to standard protocol to obtain cDNA. cDNA samples were PCR amplified using above conditions with custom M13-tagged primers (Supplementary Table S7), and resulting PCR products were purified and directly sequenced using universal M13 forward and reverse primers. Analysis of Sanger sequencing data was performed using Mutation Surveyor software package (SoftGenetics, State College, PA).

### Pyrosequencing to test for KRAS/BRAF hotspot mutations

Pyrosequencing assays were designed using the PSQ Assay Design software (QIAGEN, Chatsworth, CA) to test for hotspot mutations in KRAS (codons 12, 13, 61, and 146) and BRAF (codon 600). For each assay, one of the PCR primers was biotinylated at the 5′ end and purified using high performance liquid chromatography. Primer sequences are as follows. KRAS codons 12 and 13: For 5′- TCGATGGAGGAGTTTGTAAATGA-3′, Rev 5′- biotin-TTCGTCCACAAAATGATTCTGA-3′, Seq 5′-CTTGTGGTAGTTGGAGC-3′; KRAS codon 61: For 5′- CAGACTGTGTTTCTCCCTTCTCA-3′, Rev 5′- biotin-TCCTCATGTACTGGTCCCTCATTG-3′, Seq 5′- ATATTCTCGACACAGCAG-3′; KRAS codon 146: For 5′-AGGCTCAGGACTTAGCAAGAAGTT-3′, Rev 5′-biotin-GCCCTCTCAAGAGACAAAAACAT-3′, Seq 5′-AATTCCTTTTATTGAAACAT-3′. BRAF codon 600: For 5′- TTCATGAAGACCTCACAGTAAAAA-3′, Rev 5′- biotin-CCACAAAATGGATCCAGACA-3′, Seq 5′- TGATTTTGGTCTAGCTACA-3′. All PCR reactions were performed using FastStart Taq (Roche) and primer concentrations of 0.2 uM. Cycling conditions included an initial denaturation step at 95 C for 4 min, and 49 cycles of 95 for 15 s, 54 C for 30 s, and 72 C for 20 s. Following PCR, amplification products were sequenced on a PyroMark MD pyrosequencing instrument (QIAGEN) and mutation analysis was conducted as previously described ^4^.

Sanger sequencing was used to confirm all mutations detected by pyrosequencing analysis. Isolated genomic DNA from tumor samples was used for PCR amplification of regions encompassing codons 12, 13, 61, and 146 of KRAS, and codon 600 of BRAF. Forward and reverse primers used for PCR amplification were tagged with a 5′ M13 forward (5′-GTAAAACGACGGCCAGT-3′) and 5′ M13 reverse (5′-CAGGAAACAGCTATGAC-3′) universal primer sequence, respectively. Primer sequences were as follows: KRAS codons 12 and 13: For 5′-TGGTGGAGTATTTGATAGTGTA-3′, Rev 5′- CATGAAAATGGTCAGAGAA-3′; KRAS codon 61: For 5′- TCCAGACTGTGTTTCTCCCT-3′, Rev 5′- AACCCACCTATAATGGTGAATATCT-3′; KRAS codon 146: For 5′-AGAAGCAATGCCCTCTCAAG-3′, Rev 5′-GGACTCTGAAGATGTACCTATGGTC-3′ BRAF codon 600: For 5′- TCATAATGCTTGCTCTGATAGGA-3′, Rev 5′-GGCCAAAAATTTAATCAGTGGA-3′. All reactions were carried out using 0.4 uM concentration of each primer and FastStart Taq polymerase (Roche, Indianapolis, IN). Cycling conditions for all primer pairs consisted of an initial denaturation at 95 C for 4 min followed by 39 cycles of 95 C for 30 s, 58 C for 30 s, 72 C for 30 s, and a final elongation at 72 C for 3 min.

**Mutual Exclusivity Evaluation**

To test if mutations affecting the glycosylation genes occur in a mutually exclusive fashion with respect to other known oncogenic driver mutations in CRCs (KRAS, BRAF), we applied CoMEt ^17^, which employs an exact statistical test for mutual exclusivity that has been shown to be more sensitive in detecting mutually exclusive events within combinations containing rare alterations.

**Generation of expression constructs encoding wild-type or mutant versions of candidate genes**

Full length cDNA fragments, without the C-terminal stop codon, encoding wild-type (WT) human B3GNT2, ST6GALNAC2 and B4GALT2 transcripts were PCR amplified from total RNA isolated from a reference normal colon sample. cDNA fragments encoding respective mutant alleles were generated either by direct PCR amplification of cDNA from respective colon cancer samples, or by site directed mutagenesis (QuikChange Lightning, Agilent Technologies, Santa Clara, CA) of the wild-type allele. For B3GNT2 and B4GALT2, full-length PCR products were cloned using the TA-cloning method into the *pcDNA3.1*/V5-His TOPO cloning vector (Life technologies), in frame with C-terminal V5 and His6 epitope tags. For ST6GALNAC2, PCR products corresponding to aa 29-374 were cloned into a modified SV40 promoter-driven *pZeoSV2* vector (*pIHV*) (Life technologies) that contains an insulin secretion signal to direct the secretion of the recombinant protein into the cell culture medium, and an N-terminal His6 and V5 epitope tags to facilitate purification and detection of the recombinant protein ^18^. All constructs were verified by Sanger sequencing.

**Plasmid DNA transfection**

Transfection of plasmid DNA was performed using Lipofectamine 2000 transfection reagent (Life technologies) according to recommended protocol. Briefly, 10^6^ COS7 cells (American Type Culture Collection) grown in DMEM (Life technologies) were plated in 100mm dish for 24hr before transfection, and incubated in 5% CO2 at 37 °C overnight. 4μg of plasmid DNA in 10μl of lipofectamine 2000 reagent was used per 100-mm dish.

**Recombinant protein purification**

COS7 cells were transfected with respective wild-type or mutant expression constructs. For *pcDNA3.1* constructs, the cell monolayers were washed twice with ice-cold PBS and incubated with lysis buffer (50mMTris, pH 7.5/150mMNaCl/1mMCaCl2/1mM MnCl2/EDTA-free protease inhibitor pellets/ 0.3% CHAPS) for 15 min on ice. After scraping, the lysates were clarified by centrifugation for 15 min at maximal speed. The recombinant protein was immunoprecipitated from the lysates using anti-V5 agarose beads (Sigma-Aldrich, St Louis, MO), and subsequently washed with wash buffer (50mMTris, pH 7.5/150mMNaCl/1mMCaCl2/1mM MnCl2/EDTA-free protease inhibitor pellets). For *pIHV* constructs, conditioned medium was collected 48hr post-transfection, and recombinant protein was immunoprecipitated from 9 mL of active culture medium using anti-V5 agarose beads (Sigma-Aldrich). The beads were subsequently processed with wash buffer as described above.

**Western blot analysis**

After immunoprecipitation, 1/10 fraction of the recombinant protein was mixed with equal volume of Laemmli sample buffer (Bio-Rad, Hercules, CA) at 95 °C for 5 min, and loaded onto a Bis-Tris SDS/4–12% polyacrylamide gel (Life technologies). After SDS/PAGE, proteins were transferred onto Immobilon-P PVDF membranes (EMD Millipore, Billerica, MA). Membranes were blocked for 1hr with 5% nonfat milk, and incubated with appropriate dilution of mouse anti-V5 antibody conjugated to horseradish peroxidase (Life technologies) to detect the V5-tagged proteins for both *pcDNA3.1* and *pIHV* constructs. Enhanced Chemiluminescence Plus (GE Healthcare-BioSciences, Pittsburg, PA) and ImageJ software (National Institutes of Health, Bethesda, MA) ^19^ were used to detect and quantitate respective protein bands.

**B3GNT2 enzyme assay**

For a summary of the activities, including donor and acceptor substrates and expected products for the transferases characterized in this work, please see Supplementary Figure S2A. B3GNT2-bound beads (100-150µl settled volume) were added to 250µl of B3GNT2 reaction buffer which contained 150mM MES buffer pH 7.5, 10mM MnCl_2_, 1.8mM UDP-GlcNAc (containing total of 100µCi of UDP-[^3^H]-GlcNAc per reaction), protease inhibitors (P8340 and P8849, Sigma-Aldrich) and 0.5mM LacNAc-PNP substrate (p-nitrophenyl 2-Acetamido-2-deoxy-4-O-(β-D-galactopyranosyl)-β-D-glucopyranoside) (Toronto Research Chemicals, Toronto, ON) or 0.5mM Lactose-PNP substrate (4-nitrophenyl-β-D-lactopyranoside) (Carbosynth Limited, West Berkshire, UK) ^20,21^. Reagent reaction mixtures were shaken at 37°C in a thermostated microplate shaker (Taitec Microincubator M-36) to maintain a suspension of beads. Aliquots of suspended beads were removed and quenched with an equal volume of 250mM EDTA and frozen for processing. After dilution an aliquot was removed for measuring radioactivity on a Beckman LS5801 scintillation counter. The remainder of the sample (2ml) was subjected to reverse-phase chromatography on short C18 Sep-Pak columns (Waters, Milford, MA) ^22^. Samples were eluted using 100% methanol and measured for radioactivity. Wild-type and mutant B3GNT2 transferase-specific activities were expressed as the ratio of total post- to total pre- Sep-Pak column DPM, and further normalized to protein levels in the corresponding Western blot. Plots demonstrating wild-type protein activity in comparison to vector control for both substrates are given in Supplementary Figure S2. Wild-type and mutant transferase assays were performed together on the same day of transferase isolation using identical donor and acceptor concentrations.

**B4GALT2 enzyme assay**

B4GALT2-bound beads (100-150µl settled volume) were incubated with ~250µl of B4GALT2 reaction buffer which contained 25mM Tris Base buffer pH 7.4, 0.2% Triton X-100, 10mM MnCl_2_, 2mM UDP-Gal (containing a total of 100µCi of UDP-[^3^H]-Gal), protease inhibitors (P8340 and P8849, Sigma-Aldrich) and 3mM GlcNAc-PNP (4-nitrophenyl-2-acetamido-2-deoxy-β-D-glucopyranoside) substrate (Carbosynth Limited) ^21,23^. Transferase reactions and processing were performed as described for B3GNT2 above. Wild-type activity as compared to vector control is shown in Supplementary Figure S2. Wild-type and mutants transferase reactions were performed together on the same day of isolation using the same donor and acceptor concentrations.

**ST6GALNAC2 enzyme assay**

ST6GALNAC2-bound beads (100-150µl settled volume) were added to ~500µl reaction mixtures contained 50mM MES buffer pH 6.0, 10mM MgCl_2_, 2mM CaCl_2_, 2mM CMP-Sialic acid (CMP-NeuNAc) (containing a total of 100µCi of CMP-[^3^H]-NeuNAc per reaction), 0.2% Sodium azide, protease inhibitors (P8340 and P8849, Sigma-Aldrich) and 5 mg/mL antartic fish antifreeze glycoprotein (AFGP) (gift of Arthur L. DeVries, Uni. Illinois at Urbana-Champaign) ^24,25^ or 5mg/ml asialofetuin (ASF) (Sigma-Aldrich) ^26^. Reaction mixtures were shaken at 35°C as described above; aliquots of suspended beads were removed and quenched with an equal volume of 250mM EDTA. After a 10-fold dilution, aliquots were removed for measuring radioactivity and the remainder of the sample was dialyzed against MW3500 dialysis tubing (Thermo Fisher, Snakeskin) or 3 ml G2 dialysis cassettes (Thermo Fisher, Slide-A-Lyzer) against 2L of distilled water for 72 hours at 4°C with continuous stirring. After dialysis, samples were lyophilized and reconstituted in 1 ml of water. Aliquots were removed for determining incorporation of 3H-NeuNAc into glycopeptide substrates. Relative transferase-specific activities were expressed as a ratio of post-dialysis counts to glycoprotein absorbance at 280 or 220nm and further normalized to transferase protein levels from Western blotting. Supplementary Figure S2 demonstrates positive wild-type activity in comparison to vector control for both substrates. Wild-type and mutant transferase assays were performed together on the same day of transferase isolation.

**Mass spectrophotometry**

COS7 cells were transfected with empty vector, wild-type or mutant expression constructs of B3GNT2 or B4GALT2. After 48 hours, cells were harvested and lysed with RIPA lysis buffer. Isolated protein was subjected to immunoprecipitation at 4°C overnight using anti-V5 agarose beads. Samples were subsequently washed with wash buffer (50mMTris, pH 7.5/150mMNaCl/1mMCaCl2/1mM MnCl2/EDTA-free protease inhibitor pellets), mixed with 30μl of Laemmli sample buffer (Bio-Rad, Hercules, CA) at 95 °C for 5 min, and loaded onto a Bis-Tris SDS/10% polyacrylamide gel (Life technologies). Gels were stained with Coomassie G250 dye for 1hr followed by destaining with destaining solution (40% acetic acid, 10% methanol), overnight at room temperature. Relevant protein bands were excised for proteomic analysis. Mass spectrometry was carried out using liquid chromatography-tandem mass spectrometry (LC-MS/MS) at the Center for Proteomics and Bioinformatics, Case Western Reserve University.

***In vitro* glycosidase assay**

V957 colon cancer cells were plated on collagen in 6-well plates. Cells were transfected the following day with 2μg of *pcDNA3.1*/V5-His/ empty vector, wild-type or A146V B4GALT2 using Lipofectamine 2000. After 48 hours, cells were harvested and lysed with RIPA lysis buffer. Isolated protein was subjected to immunoprecipitation at 4°C overnight using anti-V5 agarose beads. Subsequently, beads were washed with a wash buffer (50mMTris, pH 7.5/150mMNaCl/1mMCaCl2/1mM MnCl2/EDTA-free protease inhibitor pellets) and equal volumes of the beads from each sample were treated with either Peptide N-glycosidase F (PNGaseF; removes high mannose, hybrid and complex N-glycans) or a pan glycosidase protein deglycosylation mix (contains *O*-glycosidase, PNGase F, Neuraminidase, β1-4 Galactosidase and β-*N*-Acetylglucosaminidase that removes short *O*-glycans and most *N*-glycans) (New England Biolabs, Ipswich, MA) or left untreated at 37°C for 4 hours. Finally, the reaction mix was combined with 6X Laemmli sample buffer and Western blot analysis was performed using anti-V5 antibody to detect wild-type and mutant B4GALT2 proteins.

**Confocal Imaging**

COS7 cells were cultured on the surface of 12mm coverslips in 6-well plates to 90% confluency. Cells were transfected with 2μg of *pcDNA3.1*/V5-His/B3GNT2 using Lipofectamine 2000. After 48hr, cells were fixed with 4% paraformaldehyde (Electron microscopy sciences, Hatfield, PA) at 4°C for 15 min and permeabilized in 0.2% Triton X-100 for 5 min. Samples were incubated in Image-IT^TM^ FX signal enhancer (Life Technologies) for 30min and blocked in 10% goat serum (Life Technologies) for 15 min. Immunostaining was performed with anti-V5 antibody (Sigma-Aldrich, diluted 1:500 in blocking buffer) and anti-Giantin antibody (Abcam, Cambridge, MA, diluted 1:200 in blocking buffer) at room temperature for 1hr. Samples were incubated at room temperature for 1hr with AlexaFluor 488-conjugated goat anti-mouse (Life Technologies) and AlexaFluor 594-conjugated goat anti-rabbit (Cell Signaling, Danvers, MA) secondary antibodies, diluted 1:800 in blocking buffer. Nuclei were counterstained with DRAQ5 (Cell Signaling, diluted 1:1000 in 1XPBS) and stained cells were mounted using DABCO anti-fade mounting medium (Sigma-Aldrich). Cells and immunostaining was visualized with the appropriate filters using the Zeiss LSM 510 confocal microscope and image browser.

**Scratch wound cell migration assay**

Briefly, 4x10^4^ SW480 cells (American Type Culture Collection), grown in MEM media (Life technologies) with additional supplements, were plated in each well of an ImageLock 96-well plate (Essen Bioscience, Ann Arbor, MI). After 24hr, cells were transfected with either an empty vector, respective wild-type or mutant constructs of B3GNT2, ST6GALNAC2 and B4GALT2 using Lipofectamine 2000 transfection reagent. After 12hr, the WoundMaker^TM^ tool (Essen BioScience) was used to create an end-to-end scratch in each well. Culture media was replaced with serum-free MEM with supplements, and cell migration across the width of the scratch wound was quantified over a 48hr time-course using the automated IncuCyte ZOOM live cell kinetic imaging system (Essen BioScience).

**Statistical analyses**

Significant differences in enzyme activities and cell migration between respective wild-type and mutant versions of B3GNT2, ST6GALNAC2, and B4GALT2 were estimated using a Student’s t-test, and a P value < 0.05 was considered statistically significant.

**SUPPLEMENTARY REFERENCES**

1 Markowitz, S. *et al.* Inactivation of the type II TGF-beta receptor in colon cancer cells with microsatellite instability. *Science* **268**, 1336-1338 (1995).

2 Willson, J. K., Bittner, G. N., Oberley, T. D., Meisner, L. F. & Weese, J. L. Cell culture of human colon adenomas and carcinomas. *Cancer Res* **47**, 2704-2713 (1987).

3 Adams, M. D. *et al.* Global mutational profiling of formalin-fixed human colon cancers from a pathology archive. *Mod Pathol* **25**, 1599-1608 (2012).

4 Fecteau, R. E., Lutterbaugh, J., Markowitz, S. D., Willis, J. & Guda, K. GNAS mutations identify a set of right-sided, RAS mutant, villous colon cancers. *PLoS One* **9**, e87966 (2014).

5 Umar, A. *et al.* Revised Bethesda Guidelines for hereditary nonpolyposis colorectal cancer (Lynch syndrome) and microsatellite instability. *J Natl Cancer Inst* **96**, 261-268 (2004).

6 Bao, X. & Fukuda, M. A tumor suppressor function of laminin-binding alpha-dystroglycan. *Methods Enzymol* **479**, 387-396 (2010).

7 de Bernabe, D. B. *et al.* Loss of alpha-dystroglycan laminin binding in epithelium-derived cancers is caused by silencing of LARGE. *J Biol Chem* **284**, 11279-11284 (2009).

8 Nairn, A. V. *et al.* Regulation of glycan structures in animal tissues: transcript profiling of glycan-related genes. *J Biol Chem* **283**, 17298-17313 (2008).

9 Li, H. & Durbin, R. Fast and accurate short read alignment with Burrows-Wheeler transform. *Bioinformatics* **25**, 1754-1760 (2009).

10 Li, R. *et al.* SNP detection for massively parallel whole-genome resequencing. *Genome Res* **19**, 1124-1132 (2009).

11 McKenna, A. *et al.* The Genome Analysis Toolkit: a MapReduce framework for analyzing next-generation DNA sequencing data. *Genome Res* **20**, 1297-1303 (2010).

12 Li, H. *et al.* The Sequence Alignment/Map format and SAMtools. *Bioinformatics* **25**, 2078-2079 (2009).

13 Genomes Project, C. *et al.* An integrated map of genetic variation from 1,092 human genomes. *Nature* **491**, 56-65 (2012).

14 Sjoblom, T. *et al.* The consensus coding sequences of human breast and colorectal cancers. *Science* **314**, 268-274 (2006).

15 Dees, N. D. *et al.* MuSiC: identifying mutational significance in cancer genomes. *Genome Res* **22**, 1589-1598 (2012).

16 Hochberg, Y. & Benjamini, Y. More powerful procedures for multiple significance testing. *Stat Med* **9**, 811-818 (1990).

17 Leiserson, M. D., Wu, H. T., Vandin, F. & Raphael, B. J. CoMEt: a statistical approach to identify combinations of mutually exclusive alterations in cancer. *Genome Biol* **16**, 160 (2015).

18 Guda, K. *et al.* Inactivating germ-line and somatic mutations in polypeptide N-acetylgalactosaminyltransferase 12 in human colon cancers. *Proc Natl Acad Sci U S A* **106**, 12921-12925 (2009).

19 Schneider, C. A., Rasband, W. S. & Eliceiri, K. W. NIH Image to ImageJ: 25 years of image analysis. *Nat Methods* **9**, 671-675 (2012).

20 Togayachi, A. *et al.* Beta3GnT2 (B3GNT2), a major polylactosamine synthase: analysis of B3GNT2-deficient mice. *Methods Enzymol* **479**, 185-204 (2010).

21 Togayachi, A., Sato, T. & Narimatsu, H. Comprehensive enzymatic characterization of glycosyltransferases with a beta3GT or beta4GT motif. *Methods Enzymol* **416**, 91-102 (2006).

22 Ju, T., Cummings, R. D. & Canfield, W. M. Purification, characterization, and subunit structure of rat core 1 Beta1,3-galactosyltransferase. *J Biol Chem* **277**, 169-177 (2002).

23 Almeida, R. *et al.* A family of human beta4-galactosyltransferases. Cloning and expression of two novel UDP-galactose:beta-n-acetylglucosamine beta1, 4-galactosyltransferases, beta4Gal-T2 and beta4Gal-T3. *J Biol Chem* **272**, 31979-31991 (1997).

24 Berman, E., Allerhand, A. & DeVries, A. L. Natural abundance carbon 13 nuclear magnetic resonance spectroscopy of antifreeze glycoproteins. *J Biol Chem* **255**, 4407-4410 (1980).

25 Gerken, T. A., Owens, C. L. & Pasumarthy, M. Site-specific core 1 O-glycosylation pattern of the porcine submaxillary gland mucin tandem repeat. Evidence for the modulation of glycan length by peptide sequence. *J Biol Chem* **273**, 26580-26588 (1998).

26 Kono, M. *et al.* Redefined substrate specificity of ST6GalNAc II: a second candidate sialyl-Tn synthase. *Biochem Biophys Res Commun* **272**, 94-97 (2000).

**SUPPLEMENTARY FIGURES**

**Figure S1. Mutational status of 36 glycosylation genes and *KRAS/BRAF* in colon cancers.** Mutual exclusivity analysis using an exact statistical test (CoMEt) shows that the mutations affecting any of the 36 glycosylation genes (see Supplementary Table 4) are not independent of *KRAS/BRAF* mutations (*P* > 0.05). (*) indicates V425 as the only colon cancer sample harboring mutation in the *BRAF* gene in this dataset.

**
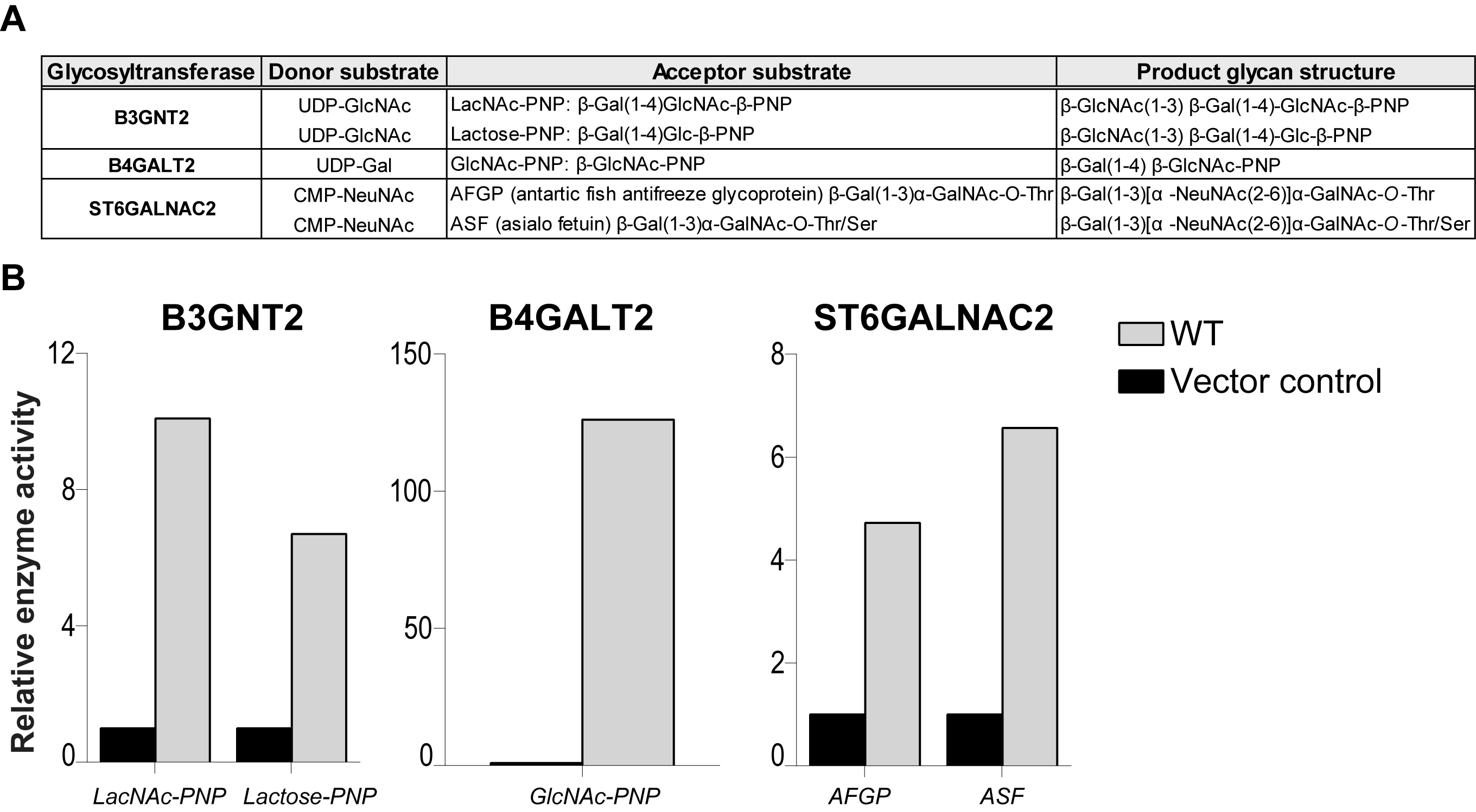
**

**Figure S2. Optimization of respective enzyme substrates.** A) Donor and acceptor substrates selected for assessing enzymatic activities of respective glycosyltransferases. B) *In vitro* enzymatic activities of wild-type B3GNT2, ST6GALNAC2 and B4GALT2 proteins against respective substrates (X-axis) at 24hr following incubation (see Methods). Fold-change in enzyme-specific activities of respective wild-type proteins, normalized to vector controls, are shown on the Y-axis. PNP refers to para-nitrophenol.

**
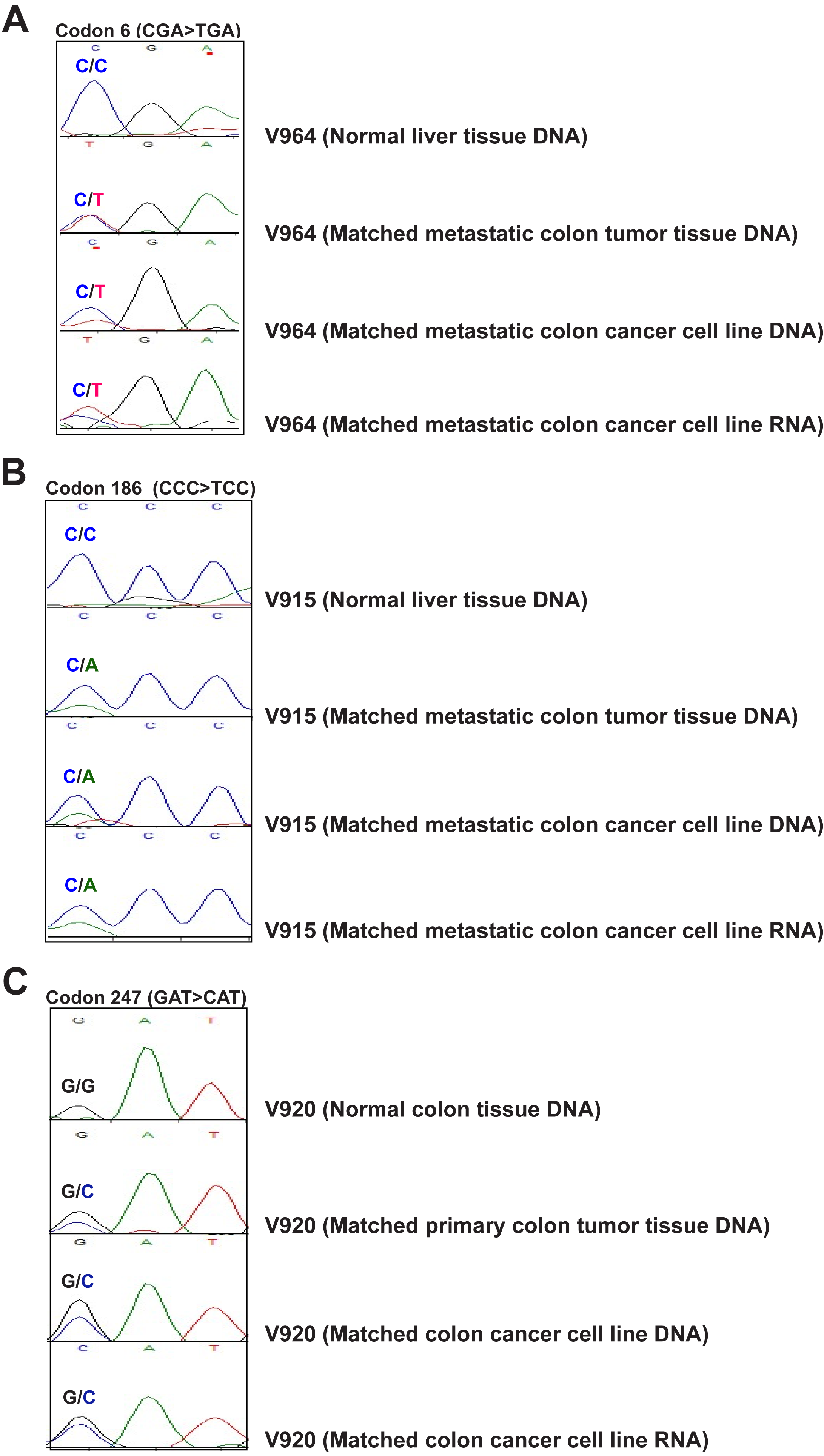
**

**Figure S3. Sanger sequencing of *B3GNT2* somatic mutations.** Representative DNA and RNA sequencing chromatograms of *B3GNT2* R6X (A), P186T (B), and D247H (C) mutations identified in respective colon cancers.

**
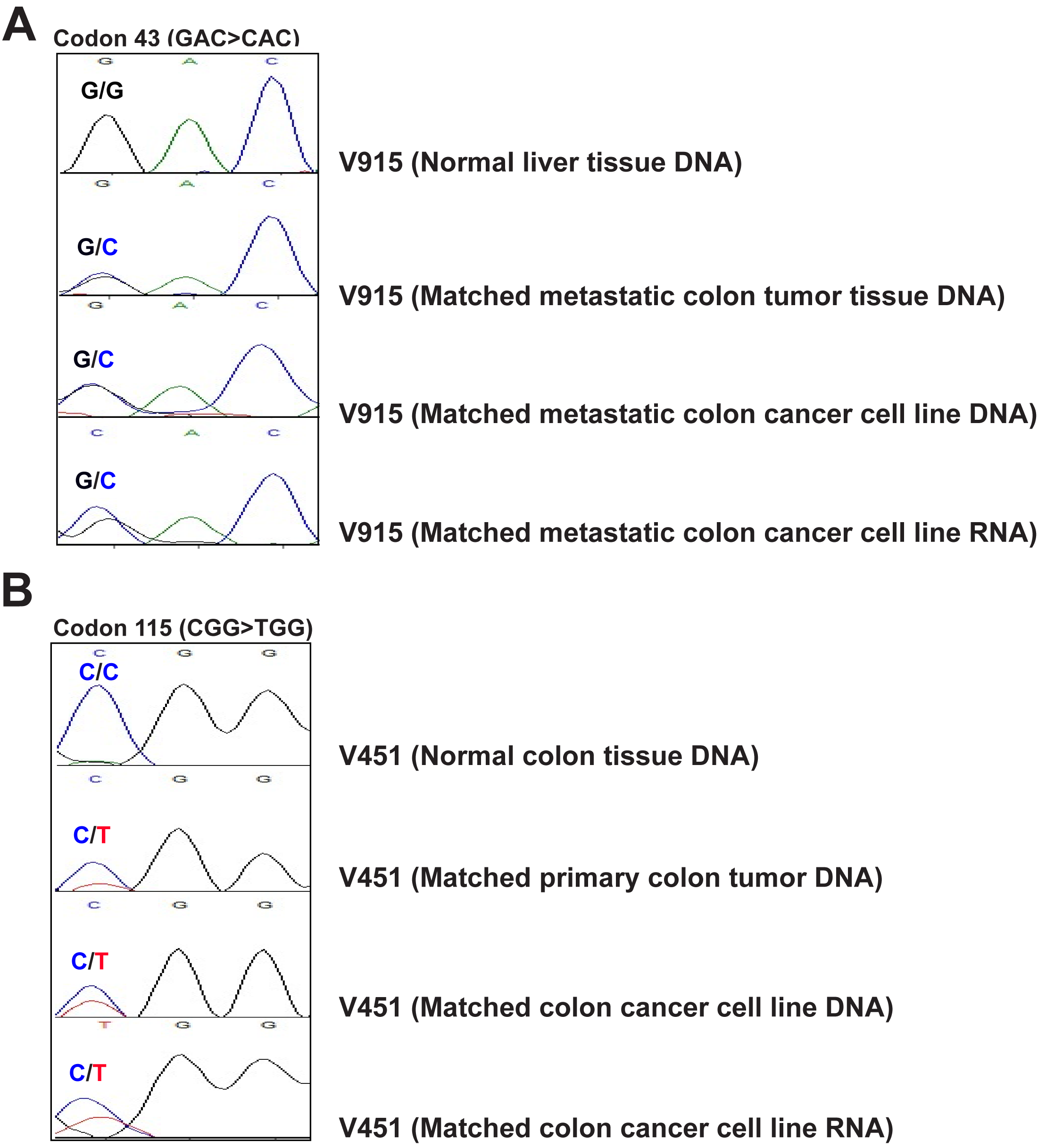
**

**Figure S4. Sanger sequencing of *ST6GALNAC2* somatic mutations.** Representative DNA and RNA sequencing chromatograms of *ST6GALNAC2* D43H (A) and R115W (B) mutations identified in respective colon cancers.


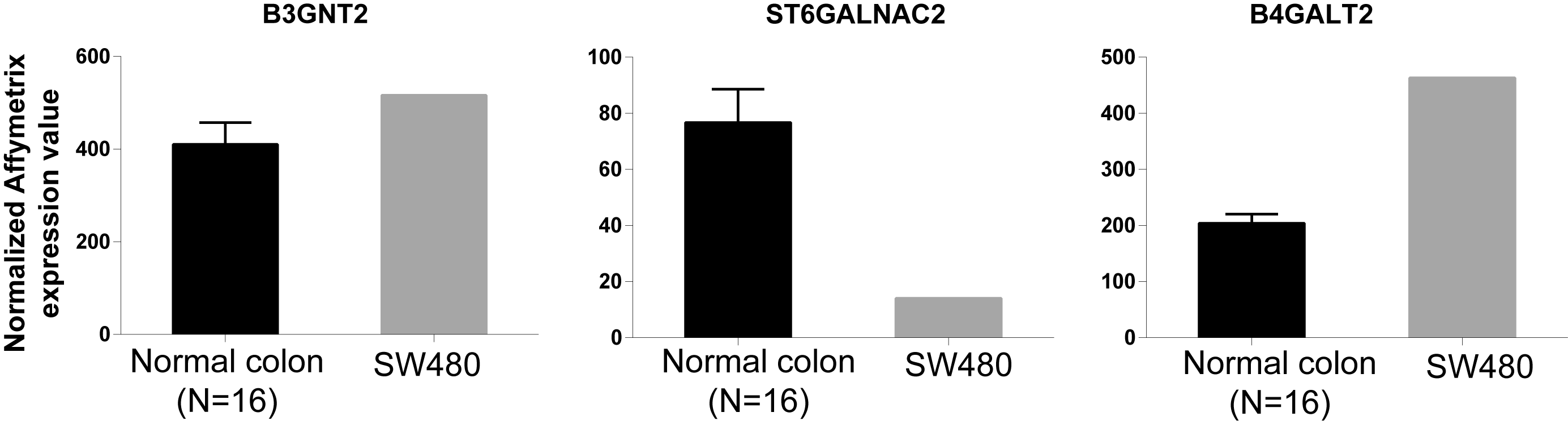
**Figure S5. Expression of B3GNT2, ST6GALNAC2, and B4GALT2 in SW480 CRC cell line.** Shown are the Affymetrix-based normalized RNA expression (Y-axis) profiles of the candidate genes in a random set of 16 normal colon epithelial RNA and the SW480 CRC parental cell line. Note the marked loss of expression of ST6GALNAC2 in SW480. Error bars in normal colon indicate mean ± s.e.m.
